# Additive neuroprotective effects of 24(S)-hydroxycholesterol and allopregnanolone in an *ex vivo* rat glaucoma model

**DOI:** 10.1038/s41598-018-31239-2

**Published:** 2018-08-27

**Authors:** Makoto Ishikawa, Takeshi Yoshitomi, Douglas F. Covey, Charles F. Zorumski, Yukitoshi Izumi

**Affiliations:** 1 Department of Ophthalmology, Akita University Graduate School of Medicine, Akita, Japan; 2 Department of Developmental Biology, Washington University School of Medicine, St. Louis, M.O USA; 3 The Taylor Family Institute for Innovative Psychiatric Research, Washington University School of Medicine, St. Louis, M.O USA; 4 Center for Brain Research in Mood Disorders, Washington University School of Medicine, St. Louis, M.O USA; 5 Department of Psychiatry, Washington University School of Medicine, St. Louis, M.O USA

## Abstract

In a rat *ex vivo* acute glaucoma model, high pressure (75 mmHg) causes swelling of ganglion cell axons and elevates levels of the endogenous steroids 24(S)-hydroxycholesterol (24SH) and allopregnanolone (AlloP). Furthermore, 24SH (0.1 µM) alone elevates AlloP levels via NMDA receptors. With this model, we now investigate possible interactions between 24SH and AlloP. We found that inhibition of AlloP synthesis with dutasteride under high pressure results in severe excitotoxicity in addition to axonal swelling. The excitotoxicity is prevented by exogenous AlloP but not 24SH, indicating that endogenous AlloP is crucial for protection. However, inhibition of 24SH synthesis with voriconazole induces severe excitotoxicity under normal pressure. Paradoxically, the excitotoxicity by voriconazole is better prevented by AlloP than 24SH. These findings suggest that inhibition of 24SH synthesis becomes excitotoxic in the absence of AlloP. We also observed that co-administration of sub-micromolar 24SH (0.1 µM) and AlloP (0.1 µM), concentrations that are only partially effective when administered alone, prevents axonal swelling under high pressure. This apparent enhanced protection indicates strong interaction between the two neurosteroids to preserve neuronal integrity, with 24SH contributing to AlloP synthesis via NMDA receptors and with AlloP playing an essential role in neuroprotection via GABA_A_ receptors.

## Introduction

Glaucoma is a leading cause of irreversible blindness^1,2^, and is characterized by apoptotic death of retinal ganglion cells (RGC). Although other factors may play a role, RGC loss is related to the level of intraocular pressure^3^. Thus, increased intraocular pressure (IOP) is a prominent risk factor, and IOP reduction is the only evidence-based treatment for glaucoma^3^. However, lowering IOP cannot halt progression of glaucoma^4–6^. Thus, it remains important to identify neuroprotective therapies aimed at reducing RGC death^7^.

**Figure 1:**
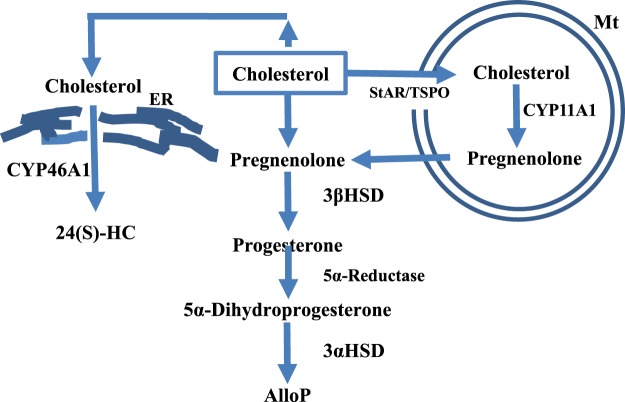
Key steps involved in the synthesis of neurosteroids. The diagram depicts biosynthetic pathways of AlloP and 24SH from cholesterol.

Cholesterol metabolism is essential for neuronal survival because impairments in synthesis and transport of cholesterol have deleterious effects on neuronal function^8–10^ and morphology^11^. In the brain, cholesterol is enzymatically converted into 24S-hydroxycholesterol (24SH) by CYP46A1, and 24SH is eliminated from the brain across the blood–brain barrier. Additionally, 27-hydroxycholesterol (27HC) and pregnenolone are synthesized from cholesterol by CYP27A1 and CYP11A1, respectively. However, under physiological conditions the amounts of 27HC and pregnenolone are much lower than 24SH^12^, suggesting that CYP46A1 is the main enzyme responsible for cholesterol metabolism in the brain. In contrast to brain, equal amounts of pregnenolone and 24SH are found in retina^13^. Because pregnenolone is a precursor of allopregnanolone (AlloP), it is likely that cholesterol is more readily converted to AlloP in the retina compared to brain (Fig. 1). Indeed, we found that pressure elevation facilitates the synthesis of AlloP in the rat *ex vivo* dissociated retina^14^. AlloP, a potent and effective positive modulator of γ-aminobutyric acid (GABA) receptors, is an endogenous protectant of RGC against pressure-induced damage, and exogenously administered AlloP (1 µM) ameliorates pressure-induced retinal injury^14,15^.

Because 24SH is a selective positive modulator of N-methyl-D-aspartate (NMDA) receptors^16^, 24SH was expected to worsen pressure-induced excitotoxicity in our *ex vivo* glaucoma model. Against this expectation, exogenously administered 24SH (1 µM) ameliorated pressure-induced retinal injury^15^. Furthermore, we observed increases in 24SH synthesis during pressure elevation suggesting a physiological role of 24SH^17^. We also found that voriconazole, an inhibitor of CYP46A1, the enzyme responsible for 24SH synthesis, is severely retinotoxic even at 10 mmHg^17^, suggesting that CYP46A1 activity and 24SH synthesis are important in preserving retinal integrity. Furthermore, full protection against voriconazole required 30 μM 24SH, a concentration that exceeds the levels needed to protect against high pressure-induced damage^17^. Taken together, the observations with AlloP and 24SH raise questions about possible interactions between 24SH and AlloP in the retina.

In the present study, we examined functional interactions between AlloP and 24SH using inhibitors of key synthetic enzymes along with various concentrations of exogenous AlloP and 24SH, alone and together, examining whether these two neurosteroids (NSs) provide additive neuroprotection in the retina.

## Results

### Combination effects of pressure elevation and inhibitors of neurosteroid synthesis

#### Effects of pressure elevation and picrotoxin

**Figure 2:**
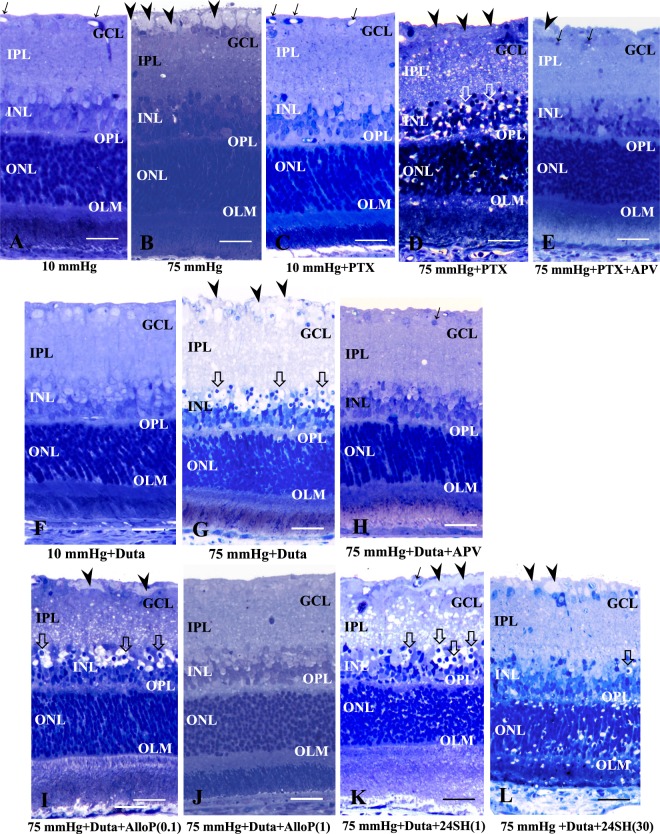
Effects of pressure elevation, picrotoxin, APV, dutasteride, and neurosteroids. (**A**,**B**) Light micrographs of pressure-loaded retinas. In retinas incubated at 10 mmHg (A), no abnormal changes were detected in any retinal layers. Arrows indicate blood vessels. Prominent swelling of optic nerve fibers (arrowheads) was observed in a retina incubated at 75 mmHg. Small vacuoles were also present in the IPL or INL. Retinal degeneration was not observed in other retinal layers (B). (**C**,**D**) Light micrographs of pressure-loaded retinas incubated with 1 μM picrotoxin (PTX) at 10 mmHg (C) and 75 mmHg (D). In retinas incubated at 10 mmHg, no abnormal changes were detected in any retinal layers (C). Administration of PTX induced excitotoxic changes characterized by bull’s eye formation in the INL (open arrows) and edematous IPL along with axonal degeneration in the NFL (arrowheads) in the retina at 75 mmHg (D). (**E**) Administration of APV (50 μM) substantially blocked excitotoxic changes in the retina incubated with 1 μM PTX at 75 mmHg. Arrows and arrowhead indicate pyknotic RGC nuclei and swollen axon, respectively. (**F**) Administration of 1 μM dutasteride (Duta) induced no remarkable changes at 10 mmHg. (**G**) Excitotoxic changes characterized by bull’s eye formation in the INL (open arrows) and edematous IPL along with axonal degeneration in the NFL (arrowheads) were observed in the retina incubated with Duta at 75 mmHg. (**H**) Administration of APV substantially blocked excitotoxic changes in the retina incubated with Duta at 75 mmHg. Arrow indicates pyknosis of the RGC nuclei. (**I**,**J**) Concentration-dependent changes in the retina incubated with AlloP at 75 mmHg. Administration of 0.1 μM AlloP did not inhibit the axonal swelling (arrowheads) or bull’s eye formation (open arrows) in the INL (**I**). AlloP exhibited substantial neuroprotection against Duta-induced neuronal damage at 1 μM (J). (**K**,**L**) Combination effects of Duta and 1 μM 24SH (K) or Duta and 30 μM 24SH (L). Administration of 1 μM 24SH or 30 μM 24SH did not inhibit the retinal degeneration induced by Duta. Note the bull’s eye formation in the INL (open arrows) and the axonal swelling in the NFL (arrowheads). A number of ganglion cells were also degenerated. Arrows indicate blood vessels. (**A**–**L**) are in the same magnification. Scale bars, 15 μm.

We initially confirmed our prior observation about the effects of pressure elevation on retinal morphology^18^. Retinas incubated at 10 mmHg for 24 hours exhibited no remarkable changes in morphology (Fig. 2A). We next examined the effects of pressure loading. Consistent with our previous report^14^, pressure elevation (75 mmHg) induced axonal swelling in the nerve fiber layer (NFL) with the other retinal layers remaining relatively intact^17^ (Fig. 2B). To determine whether GABA_A_ receptors are involved in the maintenance of retinal morphology, 1 μM picrotoxin, a GABA_A_ receptor antagonist, was administered at each pressure. Retinas incubated in the presence of picrotoxin showed no remarkable changes at 10 mmHg (Fig. 2C). By contrast, picrotoxin induced excitotoxic degeneration characterized by bull’s eye formation in the inner nuclear layer (INL) and edematous changes in the inner plexiform layer (IPL)^19^ along with axonal degeneration in the NFL under hyperbaric conditions (Fig. 2D). This excitotoxic damage was blocked by 50 µM APV, an NMDA receptor antagonist, although some nuclear degeneration in the ganglion cell layer (GCL) and axonal swelling in the NFL remained (Fig. 2E).

A quantitative assessment of structural changes induced by pressure elevation and administration of picrotoxin or APV is summarized in Supplementary Table 1 (also see Source data of Supplementary Table 1). The NFLT, NDS, and density of damaged cells in the GCL in retinas incubated with picrotoxin at 10 mmHg showed no significant changes compared to control retinas incubated at 10 mmHg. At 75 mmHg, NDS and the density of damaged cells in the GCL were greater in retinas incubated with picrotoxin compared to control retinas incubated with aCSF. The NFLT and the density of damaged cells in the GCL significantly decreased in retinas incubated with picrotoxin and APV compared to control retinas incubated with aCSF.

#### Effects of pressure elevation and dutasteride

Because of our interest in endogenous NSs, we examined the effects of 1 µM dutasteride, an effective inhibitor of AlloP synthesizing enzymes (types I and II 5α-reductase). Consistent with our previous report^14^, retinas incubated in the presence of dutasteride showed no remarkable changes at 10 mmHg (Fig. 2F). Administration of dutasteride induced severe excitotoxic retinal damage at 75 mmHg characterized by bull’s eye formation in the INL and edematous changes in the IPL^19^ along with axonal degeneration in the NFL (Fig. 2G). Administration of 50 μM APV substantially blocked excitotoxic retinal damage induced by dutasteride at 75 mmHg (Fig. 2H). Administration of 0.1 μM AlloP showed partial neuroprotection, but did not completely inhibit either dutasteride-associated retinal degeneration or axonal swelling (Fig. 2I), while 1 μM AlloP substantially inhibited the retinal damage at 75 mmHg (Fig. 2J). By contrast, we found that 1 µM 24SH failed to inhibit dutasteride-induced retinal degeneration or axonal swelling (Fig. 2K). Even at a concentration of 30 μM, 24SH failed to prevent high pressure-induced axonal swelling in the presence of dutasteride, though excitotoxic damage was attenuated (Fig. 2L).

A quantitative assessment of structural changes induced by pressure elevation and administration of dutasteride, APV, or neurosteroids is summarized in Supplementary Table 2 (also see Source data of Supplementary Table 2). The NFLT, NDS, and density of damaged cells in the GCL in retinas incubated with dutasteride at 10 mmHg showed no significant changes compared to control retinas incubated at 10 mmHg. At 75 mmHg, NDS and the density of damaged cells in the GCL significantly increased in retinas incubated with dutasteride compared to control retinas incubated with aCSF. The NFLT and the density of damaged cells in the GCL significantly decreased in retinas incubated with dutasteride and APV compared to control retinas incubated with aCSF. NDS and the density of damaged cells in the GCL significantly increased in retinas incubated with dutasteride and 0.1 μM AlloP compared to control retinas incubated with aCSF. The NFLT and the density of damaged cells in the GCL significantly decreased in retinas incubated with dutasteride and 1 μM AlloP compared to control retinas incubated with aCSF. NDS and the density of damaged cells in the GCL significantly increased in retinas incubated with dutasteride and 1 μM 24SH or with dutasteride and 30 μM 24SH compared to control retinas incubated with aCSF.

#### Effects of pressure elevation and voriconazole

**Figure 3:**
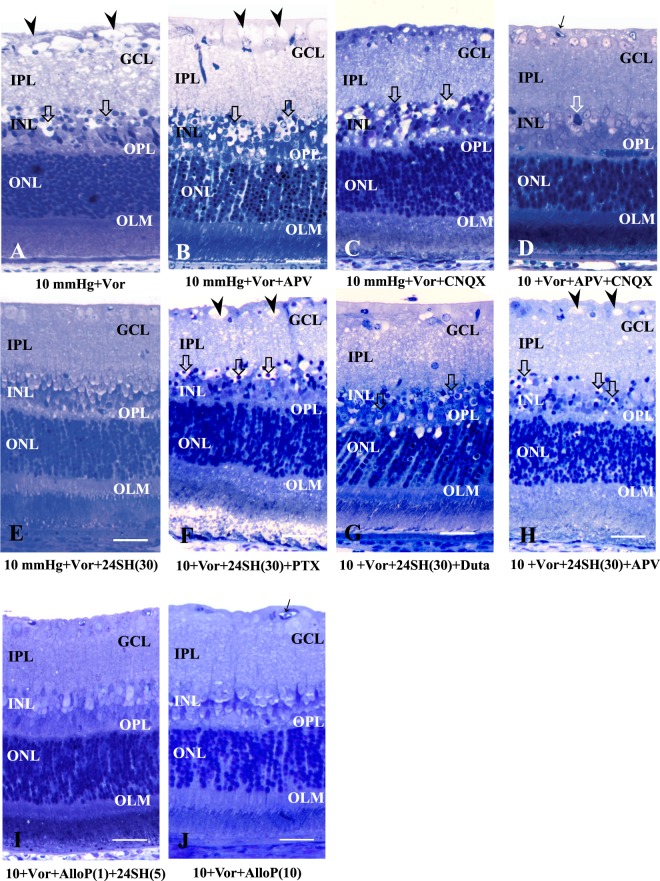
Effects of pressure elevation, voriconazole, APV, CNQX, dutasterid, and neurosteroids. (**A**) Light micrograph of the retina incubated with 10 μM voriconazole (Vot) at 10 mmHg. Administration of Vor induced excitotoxic changes characterized by bull’s eye formation in the INL (open arrows) and edematous IPL along with axonal degeneration in the NFL (arrowheads) at 10 mmHg. (**B**–**D**) Light micrographs of retinas incubated with Vor in combination with 50 μM APV alone (B), 30 μM CNQX alone (C), or 50 μM APV plus 30 μM CNQX (D) at 10 mmHg. Administration of APV alone (B) or CNQX alone (C) did not inhibit the excitotoxic degeneration induced by Vor at 10 mmHg. Arrowheads indicate degenerated axons in the NFL. Open arrows indicate the bull’s eye formation in the INL. By contrast, a combination of APV and CNQX exerted substantial neuroprotection (D). However, pyknotic degeneration in the INL (open arrow) still remained. (**E**) Administration of 30 μM 24SH exhibited substantial neuroprotection against Vor-induced neuronal damage at 10 mmHg. (**F**,**G**) Administration of 1 μM picrotoxin (PTX) (F) or 1 μM dutasteride (Duta) (G) induced bull’s eye formation in the INL (open arrows) and edematous IPL along with degeneration in the ONL in the retina incubated with Vor and 30 μM 24SH at 10 mmHg. (**H**) Administration of APV did not inhibit excitotoxic changes characterized by bull’s eye formation in the INL (open arrows) and edematous IPL along with axonal degeneration in the NFL (arrowhead) in the retina incubated with Vor and 30 μM 24SH at 10 mmHg. (**I**,**J**) Light micrographs of the retina incubated with Vor and NSs at 10 mmHg. Combination of 1 μM AlloP and 5 μM 24SH inhibited the retinal degeneration induced by Vor (I). Administration of 10 μM AlloP exerted almost complete neuroprotection (J). Arrow indicates blood vessel. (**A**–**J)** are at the same magnification. Scale bars, 15 μm.

Because we were interested in examining potential interactions between AlloP and 24SH, we also examined the effects of 10 μM voriconazole, an effective inhibitor of CYP46A1 (24SH synthesizing enzyme), on retinal morphology. We initially confirmed our prior observations that 10 μM voriconazole induced severe retinal degeneration characterized by bull’s eye formation in the INL and edematous changes in the IPL along with axonal degeneration in the NFL at 10 mmHg (Fig. 3A). Because voriconazole-induced damage has histological features similar to excitotoxicity, we also examined the effects of glutamate receptor antagonists. In retinas incubated with voriconazole at 10 mmHg, administration of either 50 μM APV (Fig. 3B), or 30 μM CNQX alone (Fig. 3C) failed to eliminate the retinal damage. However, a combination of APV and CNQX resulted in substantial neuroprotection in the presence of voriconazole (Fig. 3D), supporting the idea that voriconazole-induced damage involves activation of both types of glutamate receptor.

A quantitative assessment of structural changes induced by pressure elevation and administration of voriconazole, glutamate receptor antagonists (APV, CNQX), neurosteroids (AlloP, 24SH), picrotoxin, and dutasteride is summarized in Supplementary Table 3 (also see Source data of Supplementary Table 3). The NFLT, NDS, and the density of damaged cells in the GCL in retinas incubated with voriconazole, voriconazole plus APV, or voriconazole plus CNQX at 10 mmHg significantly increased compared to control retinas incubated at 10 mmHg. The NFLT, NDS, and the density of damaged cells in the GCL in retinas incubated with a combination of voriconazole, APV, and CNQX at 10 mmHg showed no significant changes compared to control retinas incubated at 10 mmHg.

Consistent with our previous report^17^, administration of 30 μM 24SH showed nearly complete protection against voriconazole at 10 mmHg (Fig. 3E). However, in the presence of voriconazole and 30 µM 24SH at 10 mmHg, picrotoxin (Fig. 3F) or dutasteride (Fig. 3G) induced severe excitotoxic degeneration, suggesting involvement of GABA_A_ receptors and AlloP synthesis in the neuroprotective actions of 24SH against voriconazole-induced excitotoxicity. Interestingly, APV also induced similar excitotoxic degeneration in the presence of voriconazole and 30 μM 24SH at 10 mmHg (Fig. 3H), indicating that NMDA receptor activation is necessary for 24SH to prevent the excitotoxicity.

A quantitative assessment of structural changes induced by pressure elevation and administration of voriconazole, APV, picrotoxin, dutasteride, or neurosteroids is summarized in Supplementary Table 3 (also see Source data of Supplementary Table 3).

The NFLT, NDS, and the density of damaged cells in the GCL in retinas incubated with voriconazole, 30 μM 24SH and picrotoxin at 10 mmHg significantly increased compared to control retinas incubated with voriconazole and 30 μM 24SH at 10 mmHg. The NFLT, NDS, and density of damaged cells in the GCL in retinas incubated with voriconazole, 30 μM 24SH and dutasteride at 10 mmHg significantly increased compared to control retinas incubated with voriconazole and 30 μM 24SH at 10 mmHg. The NFLT, NDS, and density of damaged cells in the GCL in retinas incubated with voriconazole, 30 μM 24SH and APV at 10 mmHg significantly increased compared to control retinas incubated with voriconazole and 30 μM 24SH at 10 mmHg.

Next, we examined whether AlloP is effective against voriconazole-induced damage. At 10 mmHg, we found that either a combination of 5 μM 24SH plus 1 μM AlloP (Fig. 3I, also see Supporting data of Fig. 3I) or administration of a high concentration of AlloP (10 μM) alone (Fig. 3J) resulted in significant neuroprotection. Neuroprotection was not observed with 5 µM 24SH alone or 1 µM AlloP alone.

A quantitative assessment of structural changes induced by pressure elevation and administration of voriconazole, APV, CNQX, or neurosteroids is summarized in Supplementary Table 3 (also see Source data of Supplementary Table 3). The NFLT, NDS, and the density of damaged cells in the GCL in retinas incubated with voriconazole, 1 μM AlloP, and 5 μM 24SH at 10 mmHg significantly decreased compared to control retinas incubated with voriconazole at 10 mmHg. The NFLT, NDS, and the density of damaged cells in the GCL in retinas incubated with voriconazole and 10 μM AlloP at 10 mmHg significantly decreased compared to control retinas incubated with voriconazole at 10 mmHg.

**Figure 4:**
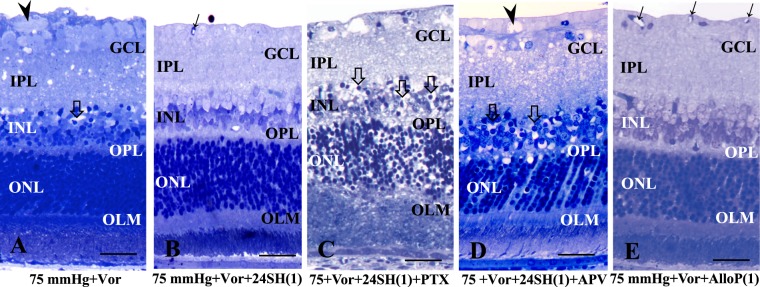
Effects of pressure elevation, voriconazole, picrotoxin, APV, and neurosteroids. (**A**) Administration with 10 μM voriconazole (Vor) induced less severe excitotoxic changes in the retina at 75 mmHg. Arrowhead indicates axonal swelling, and open arrow indicates bull’s eye formation in the INL. (**B**) Administration with 1 μM 24SH was neuroprotective in the retina incubated with Vor at 75 mmHg. Arrow indicates a blood vessel. (**C**) Administration of 1 μM picrotoxin (PTX) induced severe excitotoxic changes in a retina incubated with Vor and 1 μM 24SH at 75 mmHg. Note the degeneration in the GCL. Open arrows indicate bull’s eye formation in the INL, and open arrow indicates bull’s eye formation in the INL. (**D**) Administration of 50 μM APV did not inhibit excitotoxicity in the retina incubated with Vor and 1 μM 24SH. Arrowhead indicates degeneration in the NFL, and open arrow indicates bull’s eye formation in the INL. (**E**) Administration of 1 μM AlloP exhibited substantial neuroprotection against Vor-induced neuronal damage. Arrows indicate blood vessels. (**A**–**E**) are at the same magnification. Scale bars, 15 μm.

At 75 mmHg, voriconazole was less neurotoxic compared to 10 mmHg (Fig. 4A). Similar to 10 mmHg, administration of 1 μM 24SH also resulted in substantial protection in the presence of voriconazole at 75 mmHg (Fig. 4B). However, in the presence of voriconazole and 1 µM 24SH at 75 mmHg, picrotoxin (Fig. 4C) or APV (Fig. 4D) induced severe excitotoxic degeneration. Next, we examined whether AlloP is effective against voriconazole-induced damage; we found that administration of AlloP inhibited voriconazole-induced retinal degeneration at 1 μM (Fig. 4E), indicating that the presence of AlloP is essential in the neuroprotection.

A quantitative assessment of structural changes induced by pressure elevation and/or administration of voriconazole, APV, picrotoxin, or neurosteroids is summarized in Supplementary Table 4 (also see Source data of Supplementary Table 4). At 75 mmHg, the NFLT significantly decreased in retinas incubated with voriconazole compared to control retinas incubated with aCSF. NDS and the density of damaged cells in the GCL significantly increased in retinas incubated with voriconazole compared to control retinas incubated with aCSF. The NFLT and the density of damaged cells in the GCL significantly decreased in retinas incubated with voriconazole plus 1 μM 24SH compared to control retinas incubated with aCSF. NDS and the density of damaged cells in the GCL significantly increased in retinas incubated with voriconazole and 1 μM 24SH, plus picrotoxin, or with voriconazole and 1 μM 24SH, plus APV compared to control retinas incubated with aCSF. The NFLT and the density of damaged cells in the GCL significantly decreased in retinas incubated with voriconazole plus 1 μM AlloP compared to control retinas incubated with aCSF.

### Combination effects of AlloP and 24SH

**Figure 5:**
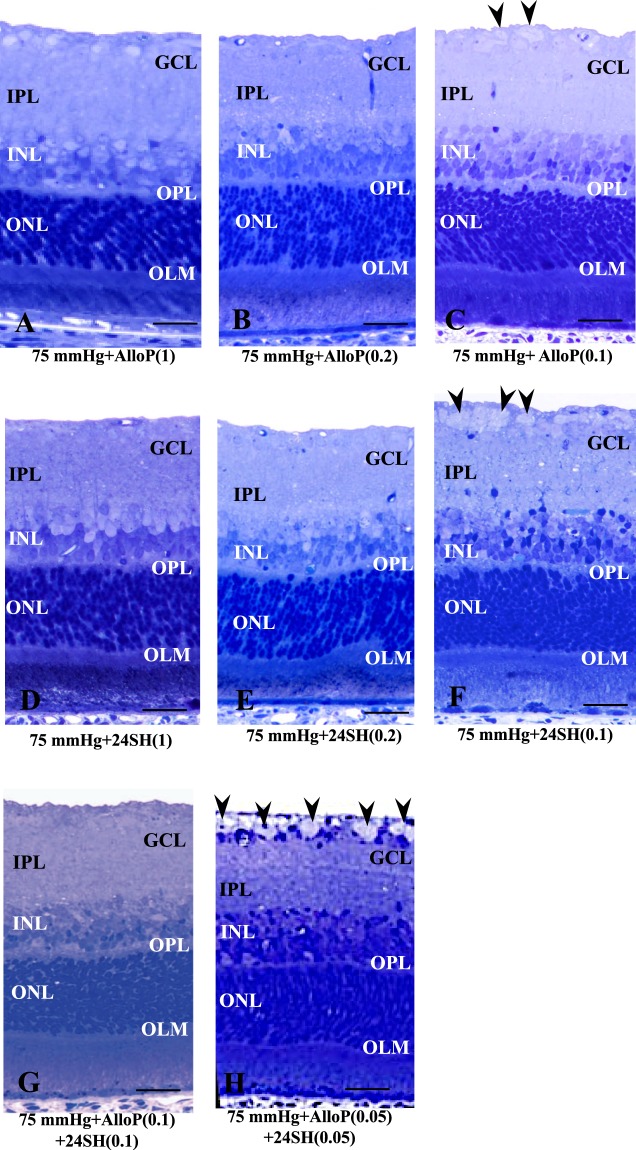
Effects of pressure elevation and neurosteroids. (**A**–**H**) Light micrographs of the middle part of the retina incubated with AlloP alone (A–C), 24SH alone (D–F), and combination with AlloP and 24SH (G–H) at 75 mmHg. (**A**–**C**) Administration of 1 μM AlloP (A) and 0.2 μM AlloP (B) substantially protected the retina from pressure-induced injury, whereas 0.1 μM AlloP (C) did not inhibit axonal swelling (arrowheads) completely. (**D**–**F**) Administration of 1 μM 24SH (D) and 0.2 μM 24SH (E) substantially protected the retina from pressure-induced injury, whereas 0.1 μM 24SH (F) did not inhibit axonal swelling (arrowheads) completely. (**G**) A combination of 0.1 μM AlloP and 0.1 μM 24SH provided complete protection. (**H**) A combination of 0.05 μM AlloP and 0.05 μM 24SH failed to inhibit pressure-induced axonal swelling (arrowheads). (**A**–**H**) are at the same magnification. Scale bars, 15 μm.

We hypothesized that there might be an interaction between AlloP and 24SH because both NSs are increased during high pressure loading and both are neuroprotective. To test this hypothesis, we applied AlloP alone, 24SH alone or a combination of AlloP and 24SH at moderate concentrations to *ex vivo* retinas at 75 mmHg. Consistent with our previous observation^14^, administration of 1 μM AlloP substantially protected the retina from axonal swelling induced by high pressure (Fig. 5A). Additionally, 0.2 μM AlloP inhibited axonal swelling induced by high pressure (Fig. 5B). However, AlloP at 0.1 μM failed to prevent axonal swelling (Fig. 5C). Also consistent with our previous observation^17^, administration of 1 μM 24SH was completely protective against high pressure (Fig. 5D). Similarly, 0.2 μM 24SH (Fig. 5E) almost completely inhibited axonal swelling, while 0.1 μM 24SH did not alter axonal swelling (Fig. 5F). Although exogenous AlloP alone or 24SH alone did not prevent axonal swelling at 0.1 μM, a combination of 0.1 μM AlloP and 0.1 μM 24SH also inhibited pressure-induced axonal swelling (Fig. 5G, also see Supporting data of Fig. 5G), indicating that these two NSs may induce additive protection at moderate concentrations. A combination of 0.05 μM AlloP and 0.05 μM 24SH failed to protect retinas from pressure-induced axonal swelling (Fig. 5H).

A quantitative assessment of nerve fiber layer thickness (NFLT), NDS, and the density of damaged cells in the GCL by a combination with AlloP and 24SH is summarized in Supplementary Table 5 (also see Source data of Supplementary Table 5). Administration of 1 μM AlloP, 0.2 μM AlloP, 1 μM 24SH, 0.2 μM AlloP, and a combination of 0.1 μM AlloP and 0.1 μM 24SH significantly decreased the NFLT, NDS, and the density of damaged cells in the GCL compared to control retinas incubated with aCSF at 75 mmHg. The NFLT and the density of damaged cells in the GCL significantly decreased in retinas incubated with 0.1 μM AlloP or with 0.1 μM 24SH compared to control retinas incubated with aCSF at 75 mmHg. The NFLT, NDS, and the density of damaged cells in the GCL showed no significant changes in retinas incubated with a combination of 0.05 μM AlloP and with 0.05 μM 24SH compared to control retinas incubated with aCSF at 75 mmHg.

### Effects of pressure elevation and neurosteroids on optic nerve head morphology

**Figure 6:**
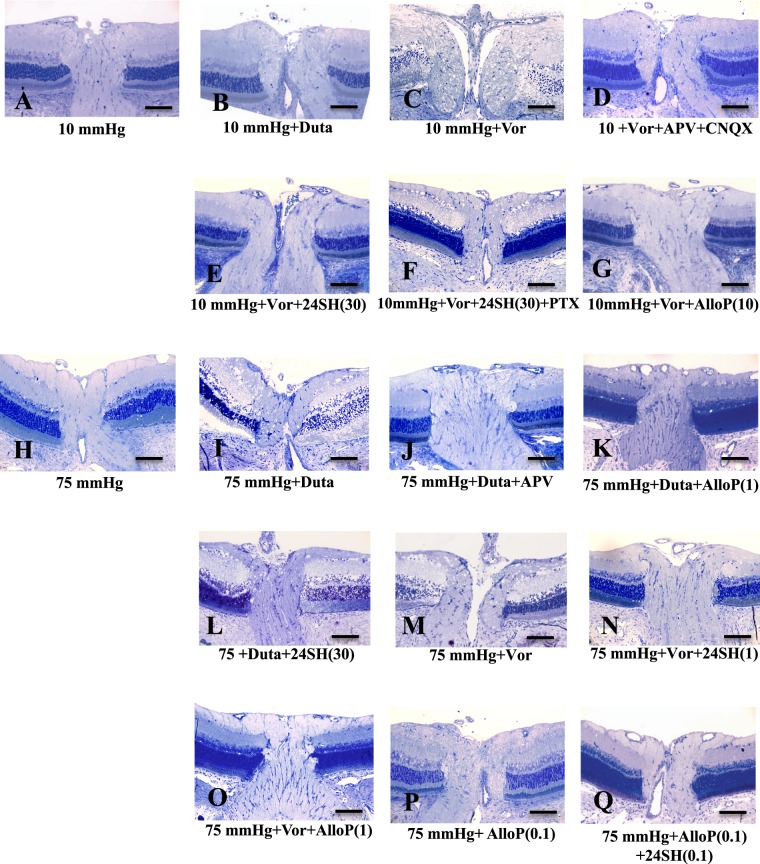
Effects of pressure elevation, dutasteride, voriconazole, APV, CNQX, and neurosteroids in optic discs. (**A**) A control retina incubated at 10 mmHg. Unmyelinated fibers run through the scleral canal, and converge into the optic nerve. (**B**) Administration of 1 μM dutasteride (Duta) induced no remarkable changes at 10 mmHg. (**C**) Administration of 10 μM voriconazole (Vor) induced severe degeneration in the optic disc at 10 mmHg. (**D**) A combination of 50 μM APV and 30 μM CNQX exerted substantial neuroprotection in the optic disc incubated with Vor. (**E**) Administration of 30 μM 24SH exhibited substantial neuroprotection against Vor-induced neuronal damage at 10 mmHg. (**F**) Administration of 1 μM picrotoxin (PTX) induced severe degeneration in optic nerve head incubated with Vor and 30 μM 24SH at 10 mmHg. (**G**) Administration of 10 μM AlloP exerted substantial neuroprotection in the optic disc incubated with Vor at 10 mmHg. (**H**) After pressure loading (75 mm Hg), the axons in the optic nerve head were swollen. (**I**) Administration of Duta induced severe degeneration in the NFL and excitotoxic degeneration in the remaining layers of the retina. (**J**) APV partially blocked Duta-induced excitotoxicity at 75 mmHg. (**K**,**L**) Administration of 1 μM AlloP substantially protected the retina from Duta-induced excitotoxicity at 75 mmHg (K). However, 30 μM 24SH did not completely inhibit Duta-induced excitotoxicity at 75 mmHg (L). (**M**–**O**) Administration with Vor induced nerve fiber degeneration in the optic nerve head at 75 mmHg (M). 24SH (N) or AlloP (O) protected optic nerve heads from Vor-induced excitotoxicity at a concentration of 1 μM at 75 mmHg. (**P**) AlloP did not protect optic nerve heads from pressure-induced injury at 0.1 μM at 75 mmHg. (**Q**) A combination of 0.1 μM AlloP and 0.1 μM 24SH provided complete protection. Panels (A–Q**)** are at the same magnification. Scale bars, 130 μm.

As the optic nerve head is the region where RGC axons come together, we examined the effects of pressure elevation and neurosteroids on optic nerve heads. When incubated at 10 mmHg for 24 hours, optic discs exhibited no remarkable changes in morphology (Fig. 6A). Administration of 1 μM dutasteride also induced no remarkable changes at 10 mmHg (Fig. 6B). In contrast, administration of 10 μM voriconazole induced excitotoxic degeneration in the optic disc at 10 mmHg (Fig. 6C), while a combination of APV and CNQX protected the optic nerve head from excitotoxic degeneration induced by voriconazole (Fig. 6D). 24SH (30 μM) protected the optic nerve head from voriconazole-induced neuronal damage at 10 mmHg (Fig. 6E), while picrotoxin induced severe degeneration with voriconazole and 30 μM 24SH at 10 mmHg (Fig. 6F). AlloP (10 μM) protected the optic nerve head from voriconazole-induced degeneration at 10 mmHg (Fig. 6G).

Pressure elevation (75 mm Hg) induced axonal swelling in the optic nerve head (Fig. 6H). At 75 mmHg, dutasteride induced severe axonal degeneration and excitotoxicity in the remaining layers of the retina (Fig. 6I), while administration of APV protected the optic nerve heads from dutasteride-induced excitotoxicity (Fig. 6J). AlloP (1 μM) induced almost complete protection in optic discs incubated with dutasteride at 75 mmHg (Fig. 6K). However, 30 μM 24SH did not completely block dutasteride-induced axonal swelling and excitotoxicity at 75 mmHg (Fig. 6L). At 75 mmHg, voriconazole induced excitotoxic degeneration (Fig. 6M), which was prevented by 1 μM 24SH (Fig. 6N) or 1 μM AlloP (Fig. 6O). At 75 mmHg again, 0.1 μM AlloP alone (Fig. 6P) failed to prevent the pressure-induced injury, while a combination of 0.1 μM AlloP and 0.1 μM 24SH provided complete protection (Fig. 6Q). Taken together, the morphological changes in the optic nerve heads were consistent with retinal changes in other *ex vivo* experiments.

A quantitative assessment of structural changes induced by pressure elevation and/or dutasteride, voriconazole, glutamate receptor antagonists, picrotoxin, and neurosteroids on the NDS in the optic nerve head is summarized in Supplementary Tables 1–6 (10 mmHg) and Supplementary Tables 2–6 (75 mmHg) (also see Source data of Supplementary Tables 1–6 and Supplementary Tables 2–6). NDS showed no significant changes in retinas incubated with dutasteride or a combination of voriconazole, APV and CNQX at 10 mmHg compared to control retinas incubated with aCSF at 10 mmHg. NDS significantly increased in retinas incubated with voriconazole at 10 mmHg compared to control retinas incubated with aCSF at 10 mmHg. NDS significantly increased in retinas incubated with voriconazole, 30 μM 24SH, and picrotoxin at 10 mmHg compared to control retinas incubated with voriconazole and 30 μM 24SH at 10 mmHg. NDS showed no significant changes in retinas incubated with voriconazole and 1 μM AlloP at 10 mmHg compared to control retinas incubated with voriconazole and 30 μM 24SH at 10 mmHg.

At 75 mmHg, NDS significantly decreased in retinas incubated with dutasteride plus APV or dutasteride plus 1 μM AlloP compared to control retinas incubated with dutasteride. NDS significantly decreased in retinas incubated with voriconazole plus 1 μM 24SH or voriconazole and 1 μM AlloP at 75 mmHg compared to control retinas incubated with voriconazole at 75 mmHg. NDS significantly decreased in retinas incubated with a combination of 0.1 μM AlloP and 0.1 μM 24SH at 75 mmHg compared to control retinas incubated with 0.1 μM AlloP at 75 mmHg.

### Effects of pressure elevation on RGC survival and neuroprotection with neurosteroids

**Figure 7:**
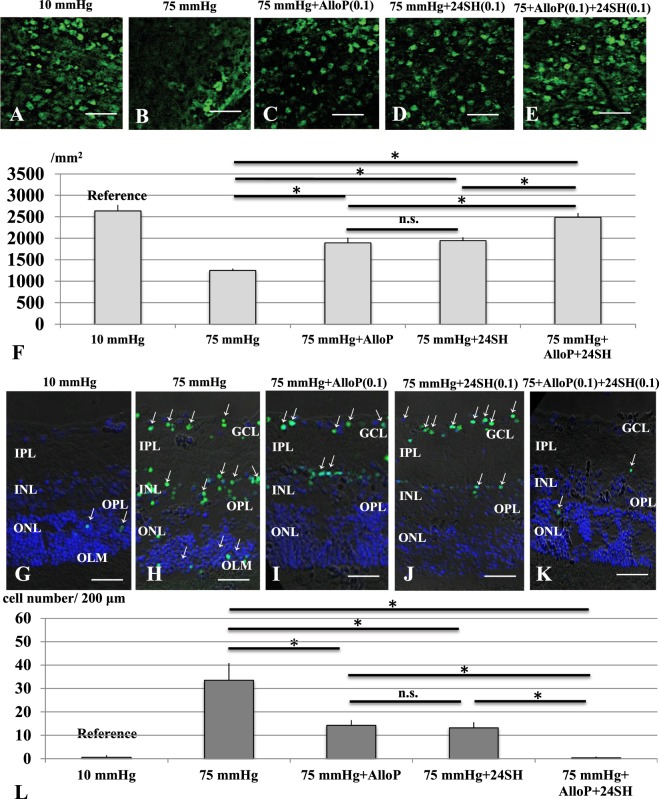
RGC survival test and visualization of apoptotic cells by fluorescence microscopy. (**A**–**E**) RGC survival using RGC marker (NeuN) in whole mount retinas. (**A**) Confocal images of NeuN-labeled RGCs in a control eye incubated at 10 mmHg. (**B**) Density of NeuN-positive RGCs was reduced at 75 mmHg compared to 10 mmHg. (**C**,**D**) Administration of AlloP (0.1 μM) alone (C) or 24SH (0.1 μM) alone (D) partially protected RGCs from pressure-induced injury. (**E**) Combination of AlloP (0.1 μM) and 24SH (0.1 μM) enhanced RGC survival under hyperbaric conditions. (**A**–**E**) are at the same magnification. Scale bars, 200 μm. (**F**) The graph presents the number of NeuN-positive cells in the whole mount retina under each experimental condition. Statistical differences were analyzed using Dunnet’s or Tukey’s multiple comparison test (*p < 0.05). (**G**–**K**) Merge of differential interference contrast images and fluorescence images using DAPI and TUNEL-fluorescent staining of retinas. (**G**) At 10 mmHg, few TUNEL-positive cells (arrows) can be observed within the ONL. (**H**) At 75 mmHg, there was a marked increase of TUNEL-positive cells in the GCL as well as some cells in the INL and ONL. (**I**,**J**) Administration of 0.1 μM AlloP alone (**I**) or 0.1 μM 24SH alone (J) significantly decreased a number of TUNEL-positive cells at 75 mmHg. (**K**) Combination of 0.1 μM AlloP and 0.1 μM 24SH almost completely inhibited apoptosis at 75 mmHg. A few TUNEL-positive cells (arrows) can be observed within the INL and ONL. a-e are at the same magnification. Scale bars, 30 μm. (**L**) The graph presents the number of TUNEL-positive RGCs per 200 μm of the retinal section. Statistical differences were analyzed using Dunnet’s or Tukey’s multiple comparison test (*p < 0.05).

In whole mounted retinas, RGCs were specifically labeled with anti-NeuN antibody (an RGC nuclear marker) at 10 mmHg (Fig. 7A). RGC damage induced by pressure elevation (75 mm Hg) was visualized as reduced numbers of cells that were positive for NeuN (Fig. 7B). An intermediate degree of protection was observed with 0.1 μM AlloP alone (Fig. 7C) or 0.1 μM 24SH alone (Fig. 7D). Compared to retinas treated with 0.1 μM AlloP alone or 0.1 μM 24SH alone, co-administration of 0.1 μM AlloP plus 0.1 μM 24SH significantly promoted survival of NeuN-positive RGCs at 75 mmHg (Fig. 7E). The graph in Fig. 7F shows the number of NeuN-positive RGCs in the retina in each condition (also see Source data of Fig. 7F).

### Pressure-induced apoptosis and neuroprotection with neurosteroids

At 10 mmHg, a small number of TUNEL-positive cells are observed only in the ONL (Fig. 7G). Exposure to elevated pressure (75 mmHg) induced apoptosis that was apparent in the GCL, INL, and ONL (Fig. 7H). An intermediate degree of protection was observed with 0.1 μM AlloP alone (Fig. 7I) or 0.1 μM 24SH alone (Fig. 7J) at 75 mmHg. Co-administration of 0.1 μM AlloP and 0.1 μM 24SH substantially inhibited apoptosis in the retina at 75 mmHg (Fig. 7K). The graph in Fig. 7L shows the number of apoptotic cells in the retina in each condition (also see Source data of Fig. 7L). These data indicate again the additive effect of the two NSs.

### LC-MS/MS analysis

**Figure 8:**
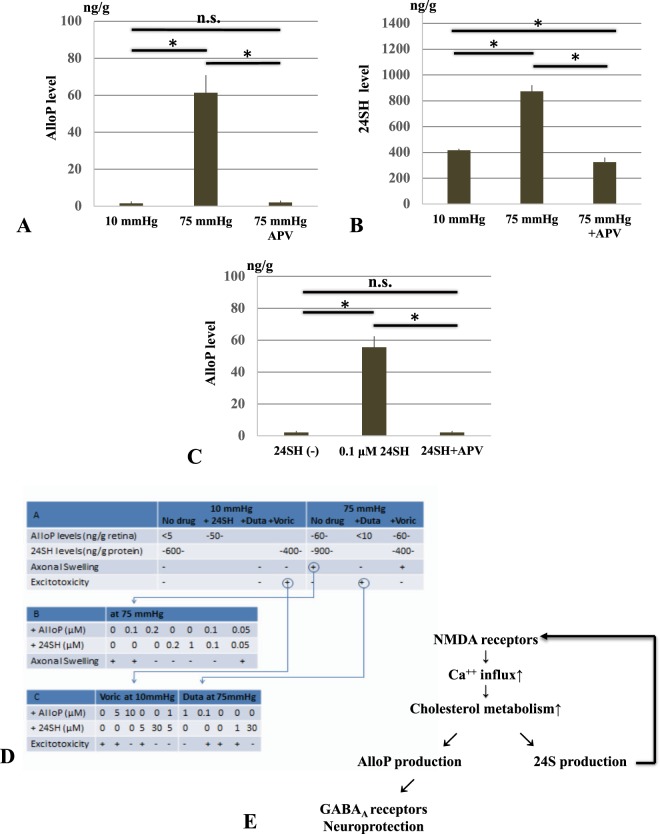
LC-MS/MS analysis of AlloP and 24SH and summary of effects of neurosteroids, their inhibitors, APV, and pressure. (**A**) Measurement of AlloP (per wet retinal weight) in retinas using LC-MS/MS. AlloP levels significantly increased at 75 mmHg (*p < 0.05) compared to control pressure (10 mmHg). Administration of 50 μM APV blocked pressure-induced AlloP increase. (**B**) Measurement of 24SH (per wet retinal weight) in retinas using LC-MS/MS. 24SH levels significantly increased at 75 mmHg (*p < 0.05) compared to control pressure (10 mmHg). Administration of APV blocked pressure-induced 24SH increase. (**C**) AlloP levels significantly increased by administration of 0.1 μM 24SH compared to the retina incubated without 24SH (*p < 0.05). Administration of APV depressed 24SH-induced AlloP increase at 10 mmHg. Statistical significance is determined by Tukey’s multiple comparison test (*p < 0.05). (**D**) Summary of current findings along with a summary of our previous observation^14,15,17^. (**D**–**A**): Effects of normobaric and hyperbaric conditions on neurosteroidogenesis, axonal swelling and excitotoxicity. In the current work, we found 24SH raises AlloP levels to about 50 ng/g at 10 mmHg. Duta: Dutasteride, Voric: voriconazole. (**D**–**B**) Current findings about effects of various combinations of AlloP and 24SH on axonal swelling at 75 mmHg. (**D**–**C**) Current findings about effects of various combinations of AlloP and 24SH on excitotoxicity by voriconazole at 10 mmHg and by dutasteride at 75 mmHg. (**E**) The diagram presents a scheme depicting how 24SH and AlloP synthesis may interact via NMDA receptors in hyperbaric conditions.

Liquid chromatography-tandem mass spectrometry (LC-MS/MS) analysis revealed that pressure elevation (75 mmHg) increased AlloP levels compared to 10 mmHg, while pressure-induced AlloP production was inhibited by 50 μM APV (Fig. 8A, also see Source data of Fig. 8A). LC-MS/MS also revealed that levels of 24SH increased in hyperbaric conditions (75 mmHg) compared to 10 mmHg, while pressure-induced 24SH production was inhibited by APV (Fig. 8B, also see Source data of Fig. 8B). These results indicate that synthesis of both 24SH and AlloP under hyperbaric conditions occurs downstream of NMDA receptor activation.

As shown above, the damage induced by voriconazole is inhibited by AlloP, but that of dutasteride is not inhibited by 24SH, implying that AlloP is more critical for neuroprotection than 24SH. We hypothesized that 24SH mobilizes membrane cholesterol to promote AlloP synthesis. To test this hypothesis, we applied 24SH (0.1 μM) to the *ex vivo* retinal specimens and determined the levels of AlloP using LC-MS/MS. At 10 mmHg, 24SH significantly increased AlloP levels. Administration of 50 μM APV inhibited the 24SH-induced AlloP increase, suggesting that the effect of 24SH on AlloP synthesis is mediated by NMDA receptor activation (Fig. 8C, also see Source data of Fig. 8C).

## Discussion

As previously reported, high pressure incubation in the *ex vivo* retina increases levels of both AlloP and 24SH. We also found that high pressure-induced axonal swelling is prevented by either of these NSs^14,15,17^. Thus, in this work, we investigated possible interactions between AlloP and 24SH to protect retinas from high pressure damage. Current findings along with a series of our previous observations are summarized in Fig. 8D.

With regard to pressure-induced axonal swelling, the present study revealed that the simultaneous administration of AlloP and 24SH is able to inhibit pressure-induced axonal swelling at sub-micromolar concentrations (Fig. 8D), resulting in apparent additive neuroprotection. Although we have already reported enhanced AlloP and 24SH synthesis in the hyperbaric condition^15,17^, the pressure-induced increase of these two NSs is not sufficient to inhibit axonal swelling in our *ex vivo* system. However, it is possible that if these two NSs are more efficiently synthesized, or if the pressure elevation is more slowly applied to the retina, then the endogenous increase in these two NSs may be sufficient for retinal protection^15^. While these two NSs are similar in terms of being induced by exposure to pressure elevation and having neuroprotective properties, these NSs appear to work in a complementary additive manner because their pharmacological actions are vastly different. While AlloP exerts neuroprotective effects, at least in part, via GABA_A_ receptors^14^, 24SH is a positive allosteric modulator of NMDA receptors^16,20^. In the present study, we found that a low concentration of 24SH is sufficient to enhance AlloP synthesis in the normobaric condition. To clarify the mechanisms of these two NSs, we examined the protective effects of 24SH and AlloP against excitotoxicity induced by dutasteride, an inhibitor of 5α-reductase, or voriconazole, an inhibitor of CYP46A1. Dutasteride is neurotoxic at 75 mmHg compared to 10 mmHg, and the neurotoxic mechanism involves excitotoxicity via activation of glutamate receptors^15^. Voriconazole also induces excitotoxicity, but is less neurotoxic at 75 mmHg compared to 10 mmHg. Whereas dutasteride-induced neurodegeneration is blocked by APV alone (Fig. 2H**)**, voriconazole-induced excitotoxicity is not blocked by APV alone (Fig. 3B) but by a combination of APV and CNQX (Fig. 3D). These disparities strongly suggest different mechanisms of retinal degeneration induced by depletion of 24SH or AlloP.

Dutasteride-induced excitotoxcity can be prevented by exogenously administered AlloP at 1 µM but not at 0.1 µM. A high concentration of 24SH (30 µM) can also attenuate this excitotoxicity but failed to prevent axonal swelling, indicating that axonal swelling and excitotoxicity involve distinct mechanisms.

In contrast to dutasteride, voriconazole induced more severe excitotoxicity in the normobaric condition than in the hyperbaric condition, and high concentrations of exogenous 24SH are required to overcome this excitotoxicity. This finding likely reflects the fact that basal AlloP levels are low in the normobaric condition. The presence of AlloP, however, is pivotal even for protection against excitotoxicity induced by voriconazole. As we hypothesized, AlloP synthesis is enhanced by administration of 24SH in the normobaric condition (Fig. 8C). However, if AlloP is not synthesized in a timely fashion or in sufficient amounts, it is unable to protect against the toxicity induced by inhibition of 24SH synthesis. Conversely, a relatively low concentration of 24SH can inhibit voriconazole-induced excitotoxicity in the presence of AlloP, while AlloP alone at a higher concentration can prevent voriconazole-induced toxicity. Thus, it appears that AlloP production is an essential mechanism for protection against voriconazole-induced excitotoxicity.

Elevated pressure simultaneously enhances synthesis of both AlloP and 24SH^14,15,17^, while 24SH induces AlloP synthesis at normal pressure. Therefore, it is possible that 24SH may contribute to induction of pressure-induced AlloP synthesis. If this is the case, AlloP production should be reduced when voriconazole is administered to inhibit 24SH synthesis. However, voriconazole actually does not inhibit AlloP synthesis in the hyperbaric condition^17^. Based on these findings, it appears that 24SH is not required to drive synthesis of AlloP in hyperbaric conditions and that other mechanisms come into play. One possibility is that more cholesterol is mobilized to produce AlloP and possibly other NSs when 24SH synthesis is blocked (Fig. 8E). It is also possible that voriconazole itself induces AlloP synthesis.

The increase of 24SH induced by pressure elevation is completely inhibited by voriconazole, while the level of 24SH in the normobaric condition is also lowered^17^. Furthermore, voriconazole is severely excitotoxic at 10 mmHg^17^. Because the normobaric AlloP level is minimal, the normobaric level of 24SH does not appear to contribute significantly to AlloP synthesis. However, the normobaric level of 24SH helps to preserve the retina even if the AlloP level is minimal, whereas the level of 24SH in the presence of voriconazole results in excitotoxicity. These observations suggest that there may be two pools of 24SH: a basal pool that persists in the presence of voriconazole and another pool that is promptly affected by voriconazole and can be mobilized acutely to provide neuroprotection in response to stressors such as pressure elevation. Taken together, our observations indicate that both AlloP and 24SH help to preserve retinal integrity in the hyperbaric condition. However, their mechanisms of neuroprotection are distinct and their actions are complimentary rather than synergistic.

Even in the normobaric condition voriconazole is not excitotoxic when AlloP is present (Fig. 3J); even without voriconazole the hyperbaric condition alone is excitotoxic when AlloP is not available (Fig. 2G). Thus, it appears that the presence of AlloP is more critical than the increase in 24SH for neuroprotection. In spite of this, dutasteride alone is not excitotoxic in the normobaric condition (Fig. 2F) and 24SH overcomes the excitotoxicity of voriconazole (Fig. 3E). However, 24SH fails to protect the retina in the presence of both voriconazole and dutasteride (Fig. 3G). This finding suggests again that AlloP is more critical for retinal protection than the increase in 24SH.

The action of AlloP is mediated via effects on GABA_A_ receptors. Similar to our previous finding that picrotoxin prevents the neuroprotective action of AlloP against dutasteride in the hyperbaric condition^14^, picrotoxin promotes voriconazole-induced excitotoxicity in the hyperbaric condition even in the presence of 24SH (Fig. 4C). This finding suggests strongly that AlloP synthesized in the hyperbaric condition works via GABA_A_ receptor activation. Furthermore, we now find that the hyperbaric condition alone is excitotoxic when GABA_A_ receptors are blocked (Fig. 2D**)**.

A key finding in this study is that AlloP synthesis by 24SH requires NMDA receptor activation (Fig. 8C), just like AlloP synthesis in the hyperbaric condition (Fig. 8A). Although 24SH is neuroprotective against voriconazole in the normobaric condition, blockade of NMDA receptors overcomes its neuroprotective action (Fig. 3H) because AlloP cannot be synthesized, even though NMDA receptor inhibition is often neuroprotective. Indeed, we found that in the hyperbaric condition APV is neuroprotective against dutasteride (Fig. 2H) and even picrotoxin (Fig. 2E). However, in the situation where AlloP synthesis is critical, blocking NMDA receptors may be paradoxically neurodegenerative. Figure 8E provides a scheme depicting possible interactions and mechanisms underlying the neuroprotection offered by 24SH and AlloP: 24SH potentiates NMDA receptors and activation of NMDA receptors triggers cholesterol metabolism^21^ resulting in AlloP synthesis and enhanced GABA receptor activation to control retinal excitability, glutamate receptors and excitotoxicity. Taken together, our results suggest that use of a combination of NSs may represent an important and novel strategy to achieve neuroprotection under glaucomatous conditions.

## Methods

Protocols for animal use were approved by the Akita University Animal Studies Committee in accordance with the guidelines of the ARVO Statement for the Use of Animals in Ophthalmic and Vision Research.

### Rat *ex vivo* eyecup preparation

Rat *ex vivo* eyecups were prepared from 28–32 day-old male Sprague-Dawley rats (Charles River Laboratories International Inc., Wilmington, MA) as previously described^18,22^. The anterior retina was carefully removed and the empty eyecup was placed on a flat cutting surface and immersed in ice-cold aCSF. The retina was not detached from the sclera. During experiments, several eyecups (3–8) were placed at the bottom of a 100 ml glass beaker filled with aCSF containing (in mM): 124 NaCl, 5 KCl, 2 MgSO4, 2 CaCl2, 1.25 NaH2PO4, 22 NaHCO3, and 10 glucose, and incubated at 30 °C for 24 hours using a closed pressure-loading system. In this model, a glass beaker was carefully placed at the bottom of an acrylic pressure chamber (2,000 ml) with pH maintained at 7.35 to 7.40. A 95% O_2_-5% CO_2_ gas mixture was delivered via plastic tubing and an air filter (Cat#SLGP033RS, Merck Millipore, Billerica, MA), with the tubing terminating 1 cm above the bottom of the beaker. Gas flow was regulated with an infusion valve and a control dial on the lid of the pressure chamber. Acutely prepared eyecups were incubated in gassed aCSF for at least 1 h at 30 °C before pressure loading. The 95% O_2_-5% CO_2_ gas mixture was infused until the pressure reading from a manometer reached the desired level. The pressure was then locked in place by adjusting the control dial of an effusion valve, and monitored continuously for 24 h at 30 °C. After maintaining the chamber at the set pressure (10 and 75 mmHg) for the indicated time, the pressure inside the chamber was carefully decreased by opening the effusion valve. The loading pressure was adjusted to intraocular pressure (IOP) in the normal condition (10 mmHg) and IOP that can occur during an acute angle closure attack (75 mmHg). In some experiments, AlloP (0.05 μM, 0.1 μM, 0.2 μM, 1 μM), 24SH (0.05 μM, 0.1 μM, 0.2 μM, 1 μM), dutasteride (a type I and II 5α-reductase inhibitor, 1 μM), voriconazole (CYP46A1 inhibitor, 10 μM), D-2-amino-5-phosphonovalerate (APV; 50 μM), and 6-cyano-7-nitroquinoxaline-2,3-dione (CNQX; 30 μM) were added to the aCSF.

The key advantages of this model include preservation of retinal morphology without ischemic degeneration induced by IOP elevation, making it possible to investigate direct effects of pressure-loading on histology and neurosteroidogenesis. Furthermore, the *ex vivo* model avoids interference from circulating steroids.

### Chemicals

AlloP was purchased from Wako Pure Chemical Industries, Ltd. (Cat#596–30841, CAS.NO 516-54-1; Osaka, Japan). Dutasteride was obtained from Adooq Bioscience LLC (Cat#A10338, CAS.NO 164656-23-9, Irvine, CA, USA). 24SH (Cat#H7037, CAS.NO 474-73-7) and voriconazole (Cat#PZ0005, CAS.NO474-73-7) were obtained from Sigma-Aldrich (St. Louis, MO). All other chemicals were purchased from Sigma-Aldrich Corp. or Nacalai Tesque (Kyoto, Japan).

### Light Microscopy

At the end of each experiment, eyecup preparations were fixed in 2.5% glutaraldehyde in 0.1 M phosphate buffer overnight at 4 °C. The fixed eyecups were rinsed in 0.1 M phosphate buffer and placed in 1% buffered osmium tetroxide for 60 minutes. The eyecups were dehydrated with an ethanol dilution series, embedded in epoxy resin (Epon 812, TAAB Laboratories, Aldermaston, UK) and cut into 1 μm thick semi-thin sections. The tissue was then stained with toluidine blue and evaluated by light microscopy.

### Data Analysis

In histological studies, we examined the middle portion of the retina, greater than 1,200 μm from the center of the optic disc along the inner limiting membrane (ILM) according to previously described methods^22^. The nerve fiber layer thickness (NFLT) was measured by light microscopy along 5–6 lines perpendicular to the pigment epithelium at a distance of 15 μm from each other around 1,200 μm from the center of the optic disc. The average NFLT was determined in 9 or 10 different light micrographs taken from 3 to 5 eyecup samples in each condition, divided by total retinal thickness, and mean ± standard deviation (SD) was analyzed and compared with control.

The severity of neuronal damage was assessed by light microscopy in 9 or 10 retinal fields or 3 optic nerve head fields from each experiment using a neuronal damage score (NDS) as previously described^18,22^. The NDS was determined in 10 different light micrographs taken from 3 to 5 eyecup samples in each condition.

The density of degenerated cells in the GCL was determined by counting 9 or 10 fields of 250 μm length at 10 different locations in light micrographs taken from the block of the middle retinal part 950 to 1450 μm from the center of the optic disc.

These morphometrical parameters were assessed by three raters, who remained unaware of the experimental condition. Upon completion of data assessment, significance of individual differences among raters was evaluated using five randomly selected samples in each morphometric parameter by one-way analysis of variance (one-way ANOVA) followed by a post-hoc test. There were no significant differences among the raters in any of the morphometric measurements.

### Immunocytochemistry

For immunocytochemistry, eyecup preparations were fixed with 4% paraformaldehyde-0.1 M phosphate buffer for 2 hours at 4 °C (5 animals per experimental group) at the end of each experiment. Eyecups were washed with ice cold PBS and incubated in blocking solution (1% donkey serum/PBS) for 2 h at 25 °C. Eyecup samples were then embedded in OCT compound (Sakura Global Holdings, Tokyo, Japan), and frozen with liquid nitrogen. Ten µm cryosections were incubated with blocking solution.

### Preparation of whole mounted retinas

The anterior part of the eye was removed by making an incision along the entire limbus. After incubation in the closed pressure system, retinas from five eyes in each group were processed for immunostaining as “whole mounted” retinas. The retina was carefully detached from the eye by making cuts around the optic nerve. Whole retinas were then flat-mounted, pinned in an acrylic plate with the RGC layer facing upward using stainless steel pins, and fixed in 4% paraformaldehyde-0.1 M phosphate buffer overnight at 4 °C. After the samples were fixed, the tissue was rinsed with PBS three times. To block nonspecific binding, the tissue was incubated in 2% BSA in PBS containing 0.5% Triton X-100. The whole mounted retinas were incubated in rabbit anti-NeuN polyclonal antibody solution (Cat#ab104225, Abcam) (1:100) by gently shaking at 4 °C overnight. After rinsing 3 times using PBS, the retina was incubated in FITC-conjugated secondary antibody (goat anti-rabbit IgG (H + L)) (Cat#81–6111, Zymed Laboratories Inc) (1:300). The retina tissue was then rinsed 3 times with PBS and mounted on glass slides using 50% PBS and 50% glycerol.

The retinal flat-mounts were imaged throughout the GCL in each of the four defined retinal quadrants 4 mm from the optic nerve head using a confocal microscope. Each quadrant was analyzed using a 1 mm^2^ frame, and counted using Image-Pro Plus software. The density of NeuN positive RGCs per square millimeter was averaged and compared in experimental and control retinas^17^.

### Apoptosis

To visualize apoptotic cells, we used the DeadEndTM Colorimetric TUNEL System (Promega, Madison, WI) to determine apoptotic cells according to the manufacturer’s instructions. The nuclei were counterstained with DAPI. After the length of each retinal section was measured (Image-Pro Plus software), the cells were counted in the whole section length and the number of cells was normalized per 200 μm of retinal section.

### LC-MS/MS

Three eyes were examined by LC-MS/MS in each condition. The measurements of AlloP and 24SH were based on previously reported methods^14,15^.

### Statistics

Data were double-checked and analyzed using a biomedical statistical computer program (http://www.gen-info.osaka-u.ac.jp/MEPHAS/dunnett.html) on a personal computer. Descriptive statistical results are presented using the mean values (mean) ± standard deviation (SD). For comparison with controls, we used Dunnett’s multiple comparison test, depending on sample numbers. For comparison with both the control and other conditions, we used Tukey’s multiple comparison test. For all analyses, p values were considered statistically significant, when the values were less than 0.05 (two-tailed).

## Electronic supplementary material

Supplementary data

Dataset 1

Dataset 2
