# Supplementary material for: Additive neuroprotective effects of 24(S)-hydroxycholesterol and allopregnanolone in an ex vivo rat glaucoma model
Source: Sci Rep. 2018 Aug 27;8:12851. doi: 10.1038/s41598-018-31239-2 (PMC6110753; doi:10.1038/s41598-018-31239-2)
Supplement: Supplementary file 1 — Supplementary data [file 41598_2018_31239_MOESM1_ESM.docx]

**Supporting data**

Title: Additive neuroprotective effects of 24(S)-hydroxycholesterol and allopregnanolone

in an ex vivo rat glaucoma model

Authors List:

^1^Makoto Ishikawa, ^1^Takeshi Yoshitomi, ^2,3^Douglas F. Covey,

^3,4,5^Charles F. Zorumski, and ^3,4,5^Yukitoshi Izumi

^1^Department of Ophthalmology,

Akita University Graduate School of Medicine, Akita, Japan

&

^2^Department of Developmental Biology,

^3^the Taylor Family Institute for Innovative Psychiatric Research,

^4^Center for Brain Research in Mood Disorders,

^5^Department of Psychiatry,

Washington University School of Medicine, St. Louis, M.O, USA.

**Supporting data of Fig. 3I.**

Light micrographs of the retina incubated with 10 μM voriconazole and 5 μM 24SH **(A)** or with 10 μM voriconazole and 1 μM AlloP **(B)** at 10 mmHg. Administration of 5 μM 24SH or 1 μM AlloP canot protect the retina from toxic effects induced by voriconazole. Note the remarkable degeneration in the GCL, IPL, and INL in **A** and **B**. **A** and **B** are in the same magnification. Scale bar, 15 μm.

**Supporting data of Fig. 5G.**

Immunohistochemical analysis of axonal swelling induced by pressure elevation and neurosteroids. **A-F.** Double immunostaining using anti-Thy 1.1 antibody (red; axonal marker of the RGC) and anti-GFAP antibody (green; glia marker). **A.** At 75 mmHg, swollen axons (arrowheads) and Muller foot plates were specifically labeled with anti-Thy 1.1 antibody and anti-GFAP antibodies, respectively. **B and C.** Labeling of swollen axons of the RGC with anti-Thy 1.1 antibody was inhibited by 1 μM AlloP (**B**). The swollen axons (arrowheads) were specifically labeled with anti-Thy 1.1 antibody in retinas incubated with 0.1 μM AlloP (**C**). **D and E.** Labeling of swollen axons of the RGC with anti-Thy 1.1 antibody was inhibited by 1 μM 24SH (**D**). The swollen axons (arrowheads) were specifically labeled with anti-Thy 1.1 antibody in retinas incubated with 0.1 μM 24SH (**E**). **F.** Labeling of swollen axons was also inhibited by a combination of 0.1 μM AlloP and 0.1 μM 24SH. The end feet of Müller cells were labeled with anti-GFAP antibody. **A to F** are at the same magnification. Scale bars, 15 μm.

**Supplementary Table 1.** Effects of pressure elevation, picrotoxin or APV on the NFLT, NDS, and density of damaged cells in the GCL.

| **Condition** | **NFLT vs. RT (%)**  **[*p*]** | **NDS**  **[*p*]** | **Damaged cells in GCL [*p*]** |
| --- | --- | --- | --- |
| **10 mmHg** | 0.8 ± 0.3  [-] | 0.1 ± 0.3  [-] | 0.1 ± 0.3  [-] |
| **10 mmHg + PTX** | 0.7 ± 0.3  [p>0.05] | 0.2 ± 0.4  [p>0.05] | 0.2 ± 0.4  [p>0.05] |
| **75 mmHg** | 11.8 ± 1.0  [-] | 0.2 ± 0.4  [-] | 15.8 ± 5.0  [-] |
| **75 mmHg + PTX** | 11.1 ± 1.5  [p>0.05] | 3.9 ± 0.3  [*p<0.05] | 58.8 ± 13.1  [*p<0.05] |
| **75 mmHg + PTX + APV** | 2.7 ± 1.7  [*p<0.05] | 0.3 ± 0.5  [p>0.05] | 3.9 ± 2.9  [*p<0.05] |

Data are mean ± SD. NFLT vs. RT (%) refers to the NFLT percentage of total retinal thickness (RT). The density of damaged cells in the GCL was counted per 250 μm of retina. P values in each parameter were calculated by Dunnett's test. PTX: picrotoxin.

**Supplementary Table 2.** Effects of pressure elevation, dutasteride, APV, AlloP, or 24SH on the NFLT, NDS, and density of damaged cells in the GCL.

| **Condition** | **NFLT vs. RT (%)**  **[*p*]** | **NDS**  **[*p*]** | **Damaged cells in GCL [*p*]** |
| --- | --- | --- | --- |
| **10 mmHg** | 0.8 ± 0.3  [-] | 0.1 ± 0.3  [-] | 0.1 ± 0.3  [-] |
| **10 mmHg + Duta** | 0.7 ± 0.2  [p>0.05] | 0.2 ± 0.4  [p>0.05] | 0.2 ± 0.4  [p>0.05] |
| **75 mmHg** | 11.8 ± 1.0  [-] | 0.2 ± 0.4  [-] | 15.8 ± 5.0  [-] |
| **75 mmHg + Duta** | 15.1 ± 6.9  [p>0.05] | 3.9 ± 0.3  [*p<0.05] | 58.8 ± 13.1  [*p<0.05] |
| **75 mmHg + Duta + APV** | 0.7 ± 0.2  [*p<0.05] | 0.2 ± 0.4  [p>0.05] | 0.4 ± 0.5  [*p<0.05] |
| **75 mmHg + Duta +**  **0.1 μM AlloP** | 7.2 ± 6.4  [p>0.05] | 2.5 ± 0.8  [*p<0.05] | 36.4 ± 16.2  [*p<0.05] |
| **75 mmHg + Duta +**  **1 μM AlloP** | 1.9 ± 0.3  [*p<0.05] | 0.2 ± 0.4  [p>0.05] | 3.0 ± 1.9  [*p<0.05] |
| **75 mmHg + Duta +**  **1 μM 24SH** | 8.0 ± 4.8  [p>0.05] | 3.5 ± 0.5  [*p<0.05] | 52.1 ± 10.1  [*p<0.05] |
| **75 mmHg + Duta +**  **30 μM 24SH** | 7.6 ± 3.1  [p>0.05] | 2.5 ± 0.5  [*p<0.05] | 35.2 ± 10.3  [*p<0.05] |

Data are mean ± SD. NFLT vs. RT (%) refers to the NFLT percentage of total retinal thickness (RT). The density of damaged cells in the GCL was counted per 250 μm of retina. P values in each parameter were calculated by Dunnett's test. Duta: dutasteride.

**Supplementary Table 3.** Effects of voriconazole, glutamate receptor antagonists, neurosteroids, picrotoxin, or dutasteride on the NFLT, NDS, and density of damaged cells in the GCL at 10 mmHg.

| **Condition** | **NFLT vs. RT (%)**  **[*p*]** | **NDS**  **[*p*]** | **Damaged cells in GCL [*p*]** |
| --- | --- | --- | --- |
| **10 mmHg** | 0.8 ± 0.5  [-] | 0.2 ± 0.4  [-] | 0.1 ± 0.3  [-] |
| **10 mmHg + Vor** | 12.2 ± 2.5  [*p<0.05] | 3.9 ± 0.3  [*p<0.05] | 36.8 ± 12.1  [*p<0.05] |
| **10 mmHg + Vor + APV** | 11.2 ± 1.2  [*p<0.05] | 3.9 ± 0.3  [*p<0.05] | 33.3 ± 6.8  [*p<0.05] |
| **10 mmHg + Vor + CNQX** | 10.4 ± 1.5  [*p<0.05] | 3.9 ± 0.3  [*p<0.05] | 33.1 ± 8.3  [*p<0.05] |
| **10 mmHg + Vor + APV+ CNQX** | 0.7 ± 0.6  [p>0.05] | 0.3 ± 0.5  [p>0.05] | 0.3 ± 0.5  [p>0.05] |
| **10 mmHg + Vor +30 μM 24SH** | 1.6 ± 0.8  [-] | 0.2 ± 0.4  [-] | 4.0 ± 2.1  [-] |
| **10 mmHg + Vor + 30 μM 24SH + PTX** | 4.6 ± 1.5  [*p<0.05] | 3.6 ± 0.5  [*p<0.05] | 27.2 ± 7.4  [*p<0.05] |
| **10 mmHg + Vor + 30 μM 24SH + Duta** | 5.1 ± 1.2  [*p<0.05] | 3.8 ± 0.4  [*p<0.05] | 27.9 ± 7.8  [*p<0.05] |
| **10 mmHg + Vor +30 μM 24SH +**  **APV** | 5.1 ± 0.8  [*p<0.05] | 3.6 ± 0.5  [*p<0.05] | 26.6 ± 5.6  [*p<0.05] |
| **10 mmHg + Vor** | 11.5 ± 1.2  [-] | 3.8 ± 0.4  [-] | 45.6 ± 12.6  [-] |
| **10 mmHg + Vor +**  **1 μM AlloP + 5 μM 24SH** | 1.6 ± 0.3  [*p<0.05] | 0.3 ± 0.5  [*p<0.05] | 2.4 ± 1.0  [*p<0.05] |
| **10 mmHg + Vor +**  **10 μM AlloP** | 1.0 ± 0.4  [*p<0.05] | 0.4 ± 0.5  [*p<0.05] | 2.1 ± 1.4  [*p<0.05] |

Data are mean ± SD. NFLT vs. RT (%) refers to the NFLT percentage of total retinal thickness (RT). The density of damaged cells in the GCL was counted per 250 μm of retina. P values in each parameter were calculated by Dunnett's test. Vor: voriconazole. Duta: dutasteride.

**Supplementary Table 4.** Effects of pressure elevation, voriconazole, APV, picrotoxin, or neurosteroids on the NFLT, NDS, and density of damaged cells in the GCL at 75 mmHg.

| **Condition** | **NFLT vs. RT (%)**  **[*p*]** | **NDS**  **[*p*]** | **Damaged cells in GCL [*p*]** |
| --- | --- | --- | --- |
| **75 mmHg** | 8.0 ± 0.8  [-] | 0.3 ± 0.5  [-] | 16.9 ± 4.6  [-] |
| **75 mmHg + Vor** | 2.3 ± 0.9  [*p<0.05] | 3.2 ± 0.8  [*p<0.05] | 57.5 ± 13.1  [*p<0.05] |
| **75 mmHg + Vor + 1 μM 24SH** | 1.2 ± 0.6  [*p<0.05] | 0.3 ± 0.5  [p>0.05] | 2.0 ± 1.7  [*p<0.05] |
| **75 mmHg + Vor + 1 μM 24SH + PTX** | 6.7 ± 2.1  [p>0.05] | 3.8 ± 0.4  [*p<0.05] | 71.5 ± 12.6  [*p<0.05] |
| **75 mmHg + Vor + 1 μM 24SH + APV** | 6.4 ± 3.9  [p>0.05] | 3.7 ± 0.5  [*p<0.05] | 71.8 ± 10.4  [*p<0.05] |
| **75 mmHg + Vor +**  **1 μM AlloP** | 1.4 ± 0.4  [*p<0.05] | 0.2 ± 0.4  [p>0.05] | 1.9 ± 1.5  [*p<0.05] |

Data are mean ± SD. NFLT vs. RT (%) refers to the NFLT percentage of total retinal thickness (RT). The density of damaged cells in the GCL was counted per 250 μm of retina. P values in each parameter were calculated by Dunnett's test. Vor: voriconazole. PTX: picrotoxin.

**Supplementary Table 5.** Effects of pressure elevation (75 mmHg) and administration of AlloP (1 μM, 0.2 μM, 0.1 μM, 0.05 μM) and 24SH (1 μM, 0.2 μM, 0.1 μM, 0.05 μM) on the NFLT, NDS, and density of damaged cells in the GCL at 75 mmHg.

| **Condition** | **NFLT vs. RT (%)**  **[*p*]** | **NDS**  **[*p*]** | **Damaged cells**  **in the GCL [*p*]** |
| --- | --- | --- | --- |
| **75 mmHg** | 11.5 ± 0.8  [-] | 0.8 ± 0.4  [-] | 17.6 ± 4.2  [-] |
| **75 mmHg + 1 μM AlloP** | 1.7 ± 0.5  [*p<0.05] | 0.1 ± 0.3  [*p<0.05] | 1.6 ± 1.3  [*p<0.05] |
| **75 mmHg + 0.2 μM AlloP** | 1.5 ± 0.4  [*p<0.05] | 0.1 ± 0.3  [*p<0.05] | 1.7 ± 1.3  [*p<0.05] |
| **75 mmHg + 0.1 μM AlloP** | 4.3 ± 1.2  [*p<0.05] | 0.3 ± 0.5  [p>0.05] | 4.4 ± 1.4  [*p<0.05] |
| **75 mmHg + 1 μM 24SH** | 1.9 ± 0.3  [*p<0.05] | 0.2 ± 0.4  [*p<0.05] | 1.7 ± 1.3  [*p<0.05] |
| **75 mmHg + 0.2 μM 24SH** | 1.8 ± 0.3  [*p<0.05] | 0.2 ± 0.4  [*p<0.05] | 1.7 ± 1.2  [*p<0.05] |
| **75 mmHg + 0.1 μM 24SH** | 5.8 ± 2.9  [*p<0.05] | 0.8 ± 0.4  [p>0.05] | 9.2 ± 2.6  [*p<0.05] |
| **75 mmHg +**  **0.1 μM AlloP + 0.1 μM 24SH** | 2.1 ± 0.4  [*p<0.05] | 0.1 ± 0.3  [*p<0.05] | 1.6 ± 0.9  [*p<0.05] |
| **75 mmHg +**  **0.05 μM AlloP + 0.05 μM 24SH** | 11.3 ± 1.2  [p>0.05] | 0.8 ± 0.4  [p>0.05] | 17.6 ± 2.7  [p>0.05] |

Data are mean ± SD. NFLT vs. RT (%) refers to the NFLT percentage of total retinal thickness (RT). The density of damaged cells in the GCL was counted per 250 μm of retina. P values in in each parameter were calculated by Dunnett's test.

**Supplementary Table 6-1.** Effects of dutasteride, voriconazole, glutamate receptor antagonists, picrotoxin, and neurosteroids on the NDS in the optic nerve head at 10 mmHg.

| **Condition** | **NDS**  **[*p*]** |
| --- | --- |
| **10 mmHg** | 0.3 ± 0.0  [-] |
| **10 mmHg + Duta** | 0.3 ± 0.0  [p>0.05] |
| **10 mmHg + Vor** | 4.0 ± 0.0  [*p<0.05] |
| **10 mmHg + Vor + APV+ CNQX** | 0.7 ± 0.7  [p>0.05] |
| **10 mmHg + Vor + 30 μM 24SH** | 0.7 ± 0.6  [-] |
| **10 mmHg + Vor + 30 μM 24SH+ PTX** | 4.0 ± 0.0  [*p<0.05] |
| **10 mmHg + Vor + 10 μM AlloP** | 0.3 ± 0.6  [p>0.05] |

Data are mean ± SD. P values in each parameter were calculated by Dunnett's test. Duta: dutasteride. Vor: voriconazole. PTX: picrotoxin.

**Supplementary Table 6-2.** Effects of dutasteride, voriconazole, APV, and neurosteroids on the NDS in the optic nerve head at 75 mmHg.

| **Condition** | **NDS**  **[*p*]** |
| --- | --- |
| **75 mmHg + Duta** | 4.0 ± 0.0  [-] |
| **75 mmHg + Duta + APV** | 1.3± 0.6  [*p<0.05] |
| **75 mmHg + Duta+ 1 μM AlloP** | 0.7 ± 0.6  [*p<0.05] |
| **75 mmHg + Vor** | 4.0 ± 0.0  [-] |
| **75 mmHg + Vor + 1 μM 24SH** | 1.0 ± 1.0  [*p<0.05] |
| **75 mmHg + Vor + 1 μM AlloP** | 0.3 ± 0.6  [*p<0.05] |
| **75 mmHg + 0.1 μM AlloP** | 1.3 ± 0.6  [-] |
| **75 mmHg + 0.1 μM AlloP+0.1 μM 24SH** | 0.0 ± 0.0  [*p<0.05] |

Data are mean ± SD. P values in each parameter were calculated by Dunnett's test or Wilcoxon-Mann-Whitney’s test. Duta: dutasteride. Vor: voriconazole.
