# Supplementary material for: Additive neuroprotective effects of 24(S)-hydroxycholesterol and allopregnanolone in an ex vivo rat glaucoma model
Source: Sci Rep. 2018 Aug 27;8:12851. doi: 10.1038/s41598-018-31239-2 (PMC6110753; doi:10.1038/s41598-018-31239-2)
Supplement: Supplementary file 2 — Dataset 1 [file 41598_2018_31239_MOESM2_ESM.docx]

**Source data 1**

Title: Additive neuroprotective effects of 24(S)-hydroxycholesterol and allopregnanolone

in an ex vivo rat glaucoma model

Authors List:

1Makoto Ishikawa, 1Takeshi Yoshitomi, 2,3Douglas F. Covey,

3,4,5Charles F. Zorumski, and 3,4,5Yukitoshi Izumi

1Department of Ophthalmology,

Akita University Graduate School of Medicine, Akita, Japan

&

2Department of Developmental Biology,

3the Taylor Family Institute for Innovative Psychiatric Research,

4Center for Brain Research in Mood Disorders,

5Department of Psychiatry,

Washington University School of Medicine, St. Louis, M.O, USA.

| **Source data of Fig. 7F. RGC survival** | | | | | | | | | | | |
| --- | --- | --- | --- | --- | --- | --- | --- | --- | --- | --- | --- |
|  | | 10 mmHg | | 75 mmHg | | 100 nM AlloP | | 100 nM 24SC | | AlloP+24SC | |
| 1 | | 2642 | | 1203 | | 1778 | | 1873 | | 2522 | |
| 2 | | 2720 | | 1298 | | 1820 | | 1910 | | 2457 | |
| 3 | | 2812 | | 1220 | | 1825 | | 1898 | | 2587 | |
| 4 | | 2435 | | 1261 | | 1990 | | 2054 | | 2341 | |
| 5 | | 2564 | | 1289 | | 2056 | | 2005 | | 2553 | |
| Average | | 2634.6 | | 1254.2 | | 1893.8 | | 1948.0 | | 2492.0 | |
| SD | | 144.6 | | 41.7 | | 121.6 | | 77.5 | | 97.0 | |
| Dunnett's test | | Reference | | vs | | *p<0.05 | | *p<0.05 | | *p<0.05 | |
| Tukey's test | | Reference | | vs | | *p<0.05 | | *p<0.05 | | *p<0.05 | |
|  | |  | |  | | vs | | p>0.05 | | *p<0.05 | |
|  | |  | |  | |  | | vs | | *p<0.05 | |
|  |  | |  | |  | |  | |  | |  |

| **Source data of Fig. 7L. Apoptosis TUNEL staining** | | | | | | | | | | | |
| --- | --- | --- | --- | --- | --- | --- | --- | --- | --- | --- | --- |
|  | | 10 mmHg | | 75 mmHg | | 100 nM AlloP | | 100 nM 24SC | | AlloP+24SC | |
| 1 | | 0 | | 41 | | 13 | | 12 | | 0 | |
| 2 | | 1 | | 30 | | 15 | | 10 | | 1 | |
| 3 | | 0 | | 35 | | 17 | | 15 | | 0 | |
| 4 | | 2 | | 21 | | 15 | | 13 | | 1 | |
| 5 | | 0 | | 32 | | 11 | | 16 | | 0 | |
| Average | | 0.6 | | 31.8 | | 14.2 | | 13.2 | | 0.4 | |
| SD | | 0.9 | | 7.3 | | 2.3 | | 2.4 | | 0.5 | |
| Dunnett's test | | Referece | | vs | | *p<0.05 | | *p<0.05 | | *p<0.05 | |
| Tukey's test | | Referece | | vs | | *p<0.05 | | *p<0.05 | | *p<0.05 | |
|  | |  | |  | | vs | | p>0.05 | | *p<0.05 | |
|  | |  | |  | |  | | vs | | *p<0.05 | |
|  |  | |  | |  | |  | |  | |  |

| **Source data of Fig. 8A. LC-MS/MS (AlloP)** | | | |
| --- | --- | --- | --- |
| Condition | aCSF | aCSF | APV |
| Pressure | 10 mmHg | 75 mmHg | 75 mmHg |
| 1 | 1.5 | 72.3 | 0.3 |
| 2 | 1.7 | 55.1 | 2.2 |
| 3 | 2.0 | 56.7 | 0.1 |
| Average | 1.6 | 61.4 | 0.9 |
| SD | 0.2 | 9.5 | 1.2 |
| Dunnet’s test | vs | *p<0.05 | p>0.05 |
| Tukeyt's test | vs | *p<0.05 | p>0.05 |
|  |  | vs | *p<0.05 |
|  |  |  |  |
| **Source data of Fig. 8B. LC-MS/MS (24SH)** | | | |
| Condition | aCSF | aCSF | APV |
| Pressure | 10 mmHg | 75 mmHg | 75 mmHg |
| 1 | 426.0 | 921.1 | 286.3 |
| 2 | 410.0 | 825.4 | 344.8 |
| 3 | 396.0 | 873.1 | 346.2 |
| Average | 415.7 | 873.2 | 325.8 |
| SD | 12.4 | 47.9 | 34.2 |
| Dunnet’s test | vs | *p<0.05 | p>0.05 |
| Tukeyt's test | vs | *p<0.05 | *p<0.05 |
|  |  | vs | *p<0.05 |

| **Source data of Fig. 8C. LC-MS/MS (AlloP)** | | | |
| --- | --- | --- | --- |
| Condition | aCSF | 0.1 μM 24SH | 0.1 μM 24SH+APV |
| Pressure | 10 mmHg | 10 mmHg | 10 mmHg |
| 1 | 1.5 | 63.4 | 0.1 |
| 2 | 2.2 | 50.6 | 2.0 |
| 3 | 0.0 | 52.6 | 0.1 |
| Average | 1.5 | 55.5 | 0.7 |
| SD | 0.8 | 6.9 | 1.1 |
| Dunnet’s test | vs | *p<0.05 | p>0.05 |
| Tukeyt's test | vs | *p<0.05 | p>0.05 |
|  |  | vs | *p<0.05 |
