# Supplementary material for: Additive neuroprotective effects of 24(S)-hydroxycholesterol and allopregnanolone in an ex vivo rat glaucoma model
Source: Sci Rep. 2018 Aug 27;8:12851. doi: 10.1038/s41598-018-31239-2 (PMC6110753; doi:10.1038/s41598-018-31239-2)
Supplement: Supplementary file 3 — Dataset 2 [file 41598_2018_31239_MOESM3_ESM.docx]

**Source data 2 (Supplementary Tables)**

Title: Additive neuroprotective effects of 24(S)-hydroxycholesterol and allopregnanolone

in an ex vivo rat glaucoma model

Authors List:

1Makoto Ishikawa, 1Takeshi Yoshitomi, 2,3Douglas F. Covey,

3,4,5Charles F. Zorumski, and 3,4,5Yukitoshi Izumi

1Department of Ophthalmology,

Akita University Graduate School of Medicine, Akita, Japan

&

2Department of Developmental Biology,

3the Taylor Family Institute for Innovative Psychiatric Research,

4Center for Brain Research in Mood Disorders,

5Department of Psychiatry,

Washington University School of Medicine, St. Louis, M.O, USA.

**Source data of Supplementary Table 1.**

| **Source data of Table 1.** | |  |  |  |  |  |  |
| --- | --- | --- | --- | --- | --- | --- | --- |
| **NFLT%** | 10 mmHg | 10 mmHg |  | **NFLT%** | 75 mmHg | 75 mmHg | 75 mmHg |
|  |  | 1 μM PTX |  |  |  | 1 μM PTX | 1 μM PTX+ 1 μM APV |
| 1 | 0.3 | 0.4 |  | 1 | 12.8 | 12.0 | 6.9 |
| 2 | 0.5 | 0.1 |  | 2 | 12.9 | 13.0 | 2.5 |
| 3 | 0.6 | 0.5 |  | 3 | 12.5 | 11.0 | 1.3 |
| 4 | 1.2 | 0.9 |  | 4 | 12.0 | 12.2 | 3.2 |
| 5 | 0.8 | 0.8 |  | 5 | 11.8 | 10.5 | 2.7 |
| 6 | 0.7 | 0.4 |  | 6 | 11.0 | 11.1 | 1.3 |
| 7 | 1.1 | 0.7 |  | 7 | 9.8 | 9.9 | 3.5 |
| 8 | 0.8 | 1.2 |  | 8 | 12.4 | 13.1 | 0.5 |
| 9 | 0.8 | 1.1 |  | 9 | 10.6 | 8.5 | 2.4 |
| 10 | 1.0 | 0.9 |  | 10 | 11.8 | 10.1 | 3.0 |
| Average | 0.8 | 0.7 |  | Average | 11.8 | 11.1 | 2.7 |
| SD | 0.3 | 0.3 |  | SD | 1.0 | 1.5 | 1.7 |
| Dunnett's test | vs | p>0.05 |  | Dunnett's test | vs | p>0.05 | *p<0.05 |

| **NDS** | 10 mmHg | 10 mmHg |  | **NDS** | 75 mmHg | 75 mmHg | 75 mmHg |
| --- | --- | --- | --- | --- | --- | --- | --- |
|  |  | 1 μM PTX |  |  |  | 1 μM PTX | 1 μM PTX+ 1 μM APV |
| 1 | 0 | 0 |  | 1 | 0 | 4 | 1 |
| 2 | 1 | 0 |  | 2 | 0 | 4 | 0 |
| 3 | 0 | 0 |  | 3 | 0 | 4 | 1 |
| 4 | 0 | 0 |  | 4 | 1 | 4 | 0 |
| 5 | 0 | 0 |  | 5 | 0 | 4 | 0 |
| 6 | 0 | 1 |  | 6 | 0 | 4 | 0 |
| 7 | 0 | 0 |  | 7 | 0 | 4 | 1 |
| 8 | 0 | 0 |  | 8 | 1 | 4 | 0 |
| 9 | 0 | 1 |  | 9 | 0 | 4 | 0 |
| 10 | 0 | 0 |  | 10 | 0 | 3 | 0 |
| Average | 0.1 | 0.2 |  | Average | 0.2 | 3.9 | 0.3 |
| SD | 0.3 | 0.4 |  | SD | 0.4 | 0.3 | 0.5 |
| Dunnett's test | vs | p>0.05 |  | Dunnett's test | vs | *p<0.05 | p>0.05 |

| **Damaged cells** | 10 mmHg | 10 mmHg |  | **Damaged cells** | 75 mmHg | 75 mmHg | 75 mmHg |
| --- | --- | --- | --- | --- | --- | --- | --- |
|  |  | 1 μM PTX |  |  |  | 1 μM PTX | 1 μM PTX+ 1 μM APV |
| 1 | 0 | 0 |  | 1 | 18 | 65 | 5 |
| 2 | 0 | 0 |  | 2 | 9 | 41 | 7 |
| 3 | 0 | 1 |  | 3 | 19 | 58 | 3 |
| 4 | 0 | 0 |  | 4 | 12 | 51 | 4 |
| 5 | 0 | 0 |  | 5 | 14 | 62 | 10 |
| 6 | 1 | 1 |  | 6 | 18 | 61 | 1 |
| 7 | 0 | 0 |  | 7 | 11 | 43 | 2 |
| 8 | 0 | 0 |  | 8 | 26 | 76 | 0 |
| 9 | 0 | 0 |  | 9 | 18 | 50 | 4 |
| 10 | 0 | 0 |  | 10 | 13 | 81 | 3 |
| Average | 0.1 | 0.2 |  | Average | 15.8 | 58.8 | 3.9 |
| SD | 0.3 | 0.4 |  | SD | 5.0 | 13.1 | 2.9 |
| Dunnett's test | vs | p>0.05 |  | Dunnett's test | vs | *p<0.05 | *p<0.05 |

**Source data of Supplementary Table 2.**

| **NFLT%** | 10 mmHg | 10 mmHg |
| --- | --- | --- |
|  |  | 1 μM Duta |
| 1 | 0.3 | 0.5 |
| 2 | 0.5 | 0.4 |
| 3 | 0.6 | 0.8 |
| 4 | 1.2 | 0.5 |
| 5 | 0.8 | 1.0 |
| 6 | 0.7 | 0.9 |
| 7 | 1.1 | 0.7 |
| 8 | 0.8 | 0.6 |
| 9 | 0.8 | 0.3 |
| 10 | 1.0 | 0.9 |
| Average | 0.8 | 0.7 |
| SD | 0.3 | 0.2 |
| Dunnett's test | vs | p>0.05 |

| **NFLT%** | 75 mmHg | 75 mmHg | 75 mmHg | 75 mmHg | 75 mmHg | 75 mmHg | 75 mmHg |
| --- | --- | --- | --- | --- | --- | --- | --- |
|  |  | 1 μM Duta | 1 μM Duta+ 50 μM APV | 1 μM Duta+ 0.1 μM AlloP | 1 μM Duta+ 1 μM AlloP | 1 μM Duta+ 1 μM 24SC | 1 μM Duta+ 30 μM 24SC |
| 1 | 12.8 | 22.0 | 0.5 | 2.0 | 2.0 | 3.2 | 8.9 |
| 2 | 12.9 | 15.0 | 0.4 | 12.8 | 1.8 | 15.0 | 5.3 |
| 3 | 12.5 | 12.0 | 0.8 | 21.9 | 2.2 | 12.0 | 9.8 |
| 4 | 12.0 | 13.2 | 0.5 | 3.0 | 2.1 | 2.2 | 3.7 |
| 5 | 11.8 | 20.5 | 1.0 | 4.2 | 1.8 | 2.5 | 5.5 |
| 6 | 11.0 | 12.3 | 0.9 | 5.3 | 2.0 | 10.4 | 13.0 |
| 7 | 9.8 | 8.4 | 0.7 | 10.3 | 1.3 | 9.9 | 3.3 |
| 8 | 12.4 | 4.1 | 0.6 | 8.5 | 1.8 | 3.9 | 9.0 |
| 9 | 10.6 | 27.7 | 0.3 | 3.0 | 1.6 | 7.7 | 7.7 |
| 10 | 11.8 | 15.6 | 0.9 | 1.3 | 1.9 | 13.3 | 10.1 |
| Average | 11.8 | 15.1 | 0.7 | 7.2 | 1.9 | 8.0 | 7.6 |
| SD | 1.0 | 6.9 | 0.2 | 6.4 | 0.3 | 4.8 | 3.1 |
| Dunnett's test | vs | p>0.05 | *p<0.05 | p>0.05 | *p<0.05 | p>0.05 | p>0.05 |

| **NDS** | 10 mmHg | 75 mmHg |
| --- | --- | --- |
|  |  | 1 μM Duta |
| 1 | 0 | 0 |
| 2 | 1 | 0 |
| 3 | 0 | 0 |
| 4 | 0 | 1 |
| 5 | 0 | 0 |
| 6 | 0 | 0 |
| 7 | 0 | 1 |
| 8 | 0 | 0 |
| 9 | 0 | 0 |
| 10 | 0 | 0 |
| Average | 0.1 | 0.2 |
| SD | 0.3 | 0.4 |
| Dunnett's test | vs | p>0.05 |

| **NDS** | 75 mmHg | 75 mmHg | 75 mmHg | 75 mmHg | 75 mmHg | 75 mmHg | 75 mmHg |
| --- | --- | --- | --- | --- | --- | --- | --- |
|  |  | 1 μM Duta | 1 μM Duta+ 0.1 μM APV | 1 μM Duta+ 100 nM AlloP | 1 μM Duta+ 1 μM AlloP | 1 μM Duta+ 1 μM 24SC | 1 μM Duta+ 30 μM 24SC |
| 1 | 0 | 4 | 0 | 2 | 0 | 4 | 2 |
| 2 | 0 | 4 | 1 | 3 | 0 | 3 | 2 |
| 3 | 0 | 4 | 0 | 2 | 1 | 4 | 3 |
| 4 | 1 | 4 | 0 | 3 | 0 | 3 | 3 |
| 5 | 0 | 4 | 0 | 1 | 0 | 3 | 2 |
| 6 | 0 | 4 | 0 | 4 | 0 | 4 | 2 |
| 7 | 0 | 4 | 0 | 2 | 1 | 4 | 3 |
| 8 | 1 | 4 | 1 | 3 | 0 | 3 | 3 |
| 9 | 0 | 4 | 0 | 3 | 0 | 4 | 2 |
| 10 | 0 | 3 | 0 | 2 | 0 | 3 | 3 |
| Average | 0.2 | 3.9 | 0.2 | 2.5 | 0.2 | 3.5 | 2.5 |
| SD | 0.4 | 0.3 | 0.4 | 0.8 | 0.4 | 0.5 | 0.5 |
| Dunnett's test | vs | *p<0.05 | p>0.05 | *p<0.05 | p>0.05 | *p<0.05 | *p<0.05 |

| **Damaged cells** | 10 mmHg | 75 mmHg |
| --- | --- | --- |
|  |  | 1 μM Duta |
| 1 | 0 | 0 |
| 2 | 0 | 0 |
| 3 | 0 | 1 |
| 4 | 0 | 0 |
| 5 | 0 | 0 |
| 6 | 1 | 0 |
| 7 | 0 | 0 |
| 8 | 0 | 0 |
| 9 | 0 | 1 |
| 10 | 0 | 0 |
| Average | 0.1 | 0.2 |
| SD | 0.3 | 0.4 |
| Dunnett's test | vs | p>0.05 |

| **Damaged cells** | 75 mmHg | 75 mmHg | 75 mmHg | 75 mmHg | 75 mmHg | 75 mmHg | 75 mmHg |
| --- | --- | --- | --- | --- | --- | --- | --- |
|  |  | 1 μM Duta | 1 μM Duta+ 0.1 μM APV | 1 μM Duta+ 100 nM AlloP | 1 μM Duta+ 1 μM AlloP | 1 μM Duta+ 1 μM 24SC | 1 μM Duta+ 30 μM 24SC |
| 1 | 18 | 65 | 1 | 57 | 4 | 49 | 35 |
| 2 | 9 | 41 | 0 | 43 | 1 | 32 | 30 |
| 3 | 19 | 58 | 0 | 37 | 3 | 58 | 25 |
| 4 | 12 | 51 | 0 | 21 | 5 | 61 | 21 |
| 5 | 14 | 62 | 1 | 18 | 2 | 48 | 53 |
| 6 | 18 | 61 | 0 | 31 | 2 | 53 | 41 |
| 7 | 11 | 43 | 0 | 26 | 3 | 57 | 37 |
| 8 | 26 | 76 | 1 | 19 | 7 | 40 | 50 |
| 9 | 18 | 50 | 1 | 63 | 1 | 65 | 31 |
| 10 | 13 | 81 | 0 | 49 | 2 | 58 | 29 |
| Average | 15.8 | 58.8 | 0.4 | 36.4 | 3.0 | 52.1 | 35.2 |
| SD | 5.0 | 13.1 | 0.5 | 16.2 | 1.9 | 10.1 | 10.3 |
| Dunnett's test | vs | *p<0.05 | *p<0.05 | *p<0.05 | *p<0.05 | *p<0.05 | *p<0.05 |

**Source data of Supplementary Table 3.**

| **NFLT** | 10 mmHg | 10 mmHg | 10 mmHg | 10 mmHg | 10 mmHg |
| --- | --- | --- | --- | --- | --- |
|  |  | 10 μM Vori | 10 μM Vori +APV | 10 μM Vori+ CNQX | 10 μM Vori+ APV+CNQX |
| 1 | 0.100 | 13.300 | 12.300 | 9.400 | 0.300 |
| 2 | 0.400 | 9.300 | 10.100 | 10.400 | 0.500 |
| 3 | 1.600 | 14.300 | 12.300 | 12.000 | 1.600 |
| 4 | 0.900 | 17.200 | 13.100 | 11.100 | 0.600 |
| 5 | 0.900 | 10.000 | 9.900 | 12.600 | 0.700 |
| 6 | 0.100 | 9.600 | 11.000 | 10.000 | 0.070 |
| 7 | 1.500 | 12.200 | 12.000 | 8.600 | 1.300 |
| 8 | 0.700 | 10.400 | 9.600 | 9.300 | 1.600 |
| 9 | 0.800 | 12.700 | 11.100 | 12.200 | 0.300 |
| 10 | 1.100 | 12.500 | 10.300 | 8.800 | 0.040 |
| Average | 0.8 | 12.2 | 11.2 | 10.4 | 0.7 |
| SD | 0.5 | 2.5 | 1.2 | 1.5 | 0.6 |
| Dunnett's test | vs | *p<0.05 | *p<0.05 | *p<0.05 | p>0.05 |

| **NFLT** | 10 mmHg | 10 mmhg | 10 mmhg | 10 mmhg |
| --- | --- | --- | --- | --- |
|  | 10 μM Vori+ 24SH(30) | 10 μM Vori+ 24SH(30)+PTX | 10 μM Vori+ 24SH(30)+Duta | 10 μM Vori+ 24SH(30)+APV |
| 1 | 1.2 | 5.100 | 4.000 | 5.100 |
| 2 | 2.1 | 5.200 | 5.300 | 5.300 |
| 3 | 1.5 | 3.000 | 4.900 | 3.700 |
| 4 | 2.2 | 4.200 | 5.900 | 4.300 |
| 5 | 3.4 | 4.200 | 3.300 | 5.000 |
| 6 | 0.8 | 5.700 | 6.700 | 4.900 |
| 7 | 0.9 | 6.300 | 4.100 | 5.300 |
| 8 | 1 | 7.000 | 6.800 | 6.800 |
| 9 | 1.2 | 2.500 | 6.200 | 4.900 |
| 10 | 1.2 | 3.200 | 4.200 | 5.500 |
| Average | 1.6 | 4.6 | 5.1 | 5.1 |
| SD | 0.8 | 1.5 | 1.2 | 0.8 |
| Dunnett's test | vs | *p<0.05 | *p<0.05 | *p<0.05 |

| **NFLT** | 10 mmHg | 10 mmHg | 10 mmHg |
| --- | --- | --- | --- |
|  | 10 μM Vori | 10 μM Vori+ AlloP(1)+24SH(5) | 10 μM Vori+ AlloP(10) |
| 1 | 11.6 | 1.6 | 1 |
| 2 | 11.2 | 1.8 | 1.2 |
| 3 | 11.1 | 1.8 | 1 |
| 4 | 11.9 | 0.7 | 0.9 |
| 5 | 11.3 | 1.8 | 0.5 |
| 6 | 8.7 | 1.6 | 2.1 |
| 7 | 13.7 | 1.8 | 1.1 |
| 8 | 11.4 | 1.6 | 0.9 |
| 9 | 11.9 | 1.8 | 0.7 |
| 10 | 12.1 | 1.7 | 1 |
| Average | 11.5 | 1.6 | 1.0 |
| SD | 1.2 | 0.3 | 0.4 |
| Dunnett's test | vs | *p<0.05 | *p<0.05 |

| **NDS** | 10 mmHg | 10 mmHg | 10 mmHg | 10 mmHg | 10 mmHg |
| --- | --- | --- | --- | --- | --- |
|  |  | 10 μM Vori | 10 μM Vori +APV | 10 μM Vori+ CNQX | 10 μM Vori+ APV+CNQX |
| 1 | 0 | 4 | 4 | 4 | 0 |
| 2 | 1 | 4 | 4 | 4 | 1 |
| 3 | 0 | 4 | 4 | 4 | 0 |
| 4 | 0 | 4 | 4 | 4 | 0 |
| 5 | 0 | 4 | 4 | 4 | 1 |
| 6 | 1 | 4 | 4 | 4 | 1 |
| 7 | 0 | 3 | 4 | 3 | 0 |
| 8 | 0 | 4 | 4 | 4 | 0 |
| 9 | 0 | 4 | 3 | 4 | 0 |
| 10 | 0 | 4 | 4 | 4 | 0 |
| Average | 0.2 | 3.9 | 3.9 | 3.9 | 0.3 |
| SD | 0.4 | 0.3 | 0.3 | 0.3 | 0.5 |
| Dunnett's test | vs | *p<0.05 | *p<0.05 | *p<0.05 | p>0.05 |

| **NFLT** | 10 mmHg | 10 mmhg | 10 mmhg | 10 mmhg |
| --- | --- | --- | --- | --- |
|  | 10 μM Vori+ 24SH(30) | 10 μM Vori+ 24SH(30)+PTX | 10 μM Vori+ 24SH(30)+Duta | 10 μM Vori+ 24SH(30)+APV |
| 1 | 0 | 4 | 4 | 3 |
| 2 | 0 | 3 | 4 | 3 |
| 3 | 1 | 4 | 3 | 4 |
| 4 | 0 | 4 | 4 | 4 |
| 5 | 0 | 3 | 3 | 4 |
| 6 | 0 | 3 | 4 | 4 |
| 7 | 1 | 4 | 4 | 3 |
| 8 | 0 | 4 | 4 | 3 |
| 9 | 0 | 3 | 4 | 4 |
| 10 | 0 | 4 | 4 | 4 |
| Average | 0.2 | 3.6 | 3.8 | 3.6 |
| SD | 0.4 | 0.5 | 0.4 | 0.5 |
| Dunnett's test | vs | *p<0.05 | *p<0.05 | *p<0.05 |

| **NDS** | 10 mmHg | 10 mmHg | 10 mmHg |
| --- | --- | --- | --- |
|  | 10 μM Vori | 10 μM Vori+ AlloP(1)+24SH(5) | 10 μM Vori+ AlloP(10) |
| 1 | 4 | 0 | 0 |
| 2 | 4 | 0 | 0 |
| 3 | 4 | 0 | 1 |
| 4 | 4 | 1 | 1 |
| 5 | 3 | 1 | 1 |
| 6 | 4 | 0 | 0 |
| 7 | 3 | 1 | 0 |
| 8 | 4 | 0 | 0 |
| 9 | 4 | 0 | 0 |
| 10 | 4 | 0 | 1 |
| Average | 3.8 | 0.3 | 0.4 |
| SD | 0.4 | 0.5 | 0.5 |
| Dunnett's test | vs | *p<0.05 | *p<0.05 |

| **Damaged cell** | 10 mmHg | 10 mmHg | 10 mmHg | 10 mmHg | 10 mmHg |
| --- | --- | --- | --- | --- | --- |
|  |  | 10 μM Vori | 10 μM Vori +APV | 10 μM Vori+ CNQX | 10 μM Vori+ APV+CNQX |
| 1 | 0 | 34 | 21 | 33 | 0 |
| 2 | 0 | 26 | 34 | 43 | 0 |
| 3 | 0 | 51 | 41 | 22 | 0 |
| 4 | 0 | 49 | 32 | 49 | 0 |
| 5 | 0 | 31 | 29 | 31 | 0 |
| 6 | 1 | 49 | 35 | 37 | 1 |
| 7 | 0 | 51 | 46 | 34 | 1 |
| 8 | 0 | 23 | 32 | 31 | 0 |
| 9 | 0 | 33 | 29 | 25 | 1 |
| 10 | 0 | 21 | 34 | 26 | 0 |
| Average | 0.1 | 36.8 | 33.3 | 33.1 | 0.3 |
| SD | 0.3 | 12.1 | 6.8 | 8.3 | 0.5 |
| Dunnett's test | vs | *p<0.05 | *p<0.05 | *p<0.05 | p>0.05 |

| **Damaged cell** | 10 mmHg | 10 mmhg | 10 mmhg | 10 mmhg |
| --- | --- | --- | --- | --- |
|  | 10 μM Vori+ 24SH(30) | 10 μM Vori+ 24SH(30)+PTX | 10 μM Vori+ 24SH(30)+Duta | 10 μM Vori+ 24SH(30)+APV |
| 1 | 4 | 18 | 35 | 20 |
| 2 | 5 | 23 | 31 | 27 |
| 3 | 1 | 21 | 36 | 38 |
| 4 | 7 | 30 | 22 | 30 |
| 5 | 4 | 24 | 26 | 28 |
| 6 | 3 | 28 | 24 | 20 |
| 7 | 5 | 31 | 28 | 30 |
| 8 | 1 | 22 | 17 | 24 |
| 9 | 7 | 44 | 19 | 21 |
| 10 | 3 | 31 | 41 | 28 |
| Average | 4.0 | 27.2 | 27.9 | 26.6 |
| SD | 2.1 | 7.4 | 7.8 | 5.6 |
| Dunnett's test | vs | *p<0.05 | *p<0.05 | *p<0.05 |

| **Damaged cell** | 10 mmHg | 10 mmHg | 10 mmHg |
| --- | --- | --- | --- |
|  | 10 μM Vori | 10 μM Vori+ AlloP(1)+24SH(5) | 10 μM Vori+ AlloP(10) |
| 1 | 43 | 3 | 1 |
| 2 | 29 | 1 | 3 |
| 3 | 39 | 3 | 2 |
| 4 | 46 | 4 | 1 |
| 5 | 64 | 2 | 2 |
| 6 | 55 | 3 | 1 |
| 7 | 33 | 2 | 5 |
| 8 | 67 | 3 | 4 |
| 9 | 41 | 2 | 1 |
| 10 | 39 | 1 | 1 |
| Average | 45.6 | 2.4 | 2.1 |
| SD | 12.6 | 1.0 | 1.4 |
| Dunnett's test | vs | *p<0.05 | *p<0.05 |

**Source data of Supplementary Table 4.**

| **NFLT** | 75 mmHg | 75 mmHg | 75 mmHg | 75 mmHg | 75 mmHg | 75 mmHg |
| --- | --- | --- | --- | --- | --- | --- |
|  |  | 10 μM Vori | 10 μM Vor i+ 24SH(1) | 10 μMVori + PTX | 10 μM Vori + APV | 10 μM Vori + AlloP(1) |
| 1 | 0.091 | 0.002 | 0.001 | 0.096 | 0.004 | 0.017 |
| 2 | 0.084 | 0.022 | 0.023 | 0.079 | 0.027 | 0.018 |
| 3 | 0.086 | 0.033 | 0.015 | 0.087 | 0.045 | 0.016 |
| 4 | 0.079 | 0.021 | 0.022 | 0.054 | 0.141 | 0.007 |
| 5 | 0.089 | 0.019 | 0.011 | 0.042 | 0.052 | 0.007 |
| 6 | 0.069 | 0.031 | 0.008 | 0.029 | 0.087 | 0.019 |
| 7 | 0.085 | 0.032 | 0.009 | 0.076 | 0.045 | 0.0128 |
| 8 | 0.079 | 0.022 | 0.011 | 0.079 | 0.067 | 0.0127 |
| 9 | 0.068 | 0.031 | 0.012 | 0.055 | 0.098 | 0.0105 |
| 10 | 0.074 | 0.018 | 0.012 | 0.072 | 0.072 | 0.0154 |
| Average | 0.080 | 0.023 | 0.012 | 0.067 | 0.064 | 0.014 |
| SD | 0.008 | 0.009 | 0.006 | 0.021 | 0.039 | 0.004 |
| % | 8.0 | 2.3 | 1.2 | 6.7 | 6.4 | 1.4 |
| SD (%) | 0.8 | 0.9 | 0.6 | 2.1 | 3.9 | 0.4 |
| Dunnett's test | vs | *p<0.05 | *p<0.05 | p>0.05 | p>0.05 | *p<0.05 |

| **NDS** | 75 mmHg | 75 mmHg | 75 mmHg | 75 mmHg | 75 mmHg | 75 mmHg |
| --- | --- | --- | --- | --- | --- | --- |
|  |  | 10 μM Vori | 10 μM Vori+ 24SH(1) | 10 μMVori + PTX | 10 μM Vori + APV | 10 μM Vori+ AlloP(1) |
| 1 | 1 | 3 | 0 | 3 | 4 | 0 |
| 2 | 0 | 2 | 0 | 4 | 4 | 0 |
| 3 | 0 | 2 | 0 | 4 | 3 | 0 |
| 4 | 0 | 4 | 0 | 4 | 4 | 1 |
| 5 | 1 | 4 | 1 | 4 | 4 | 0 |
| 6 | 0 | 4 | 0 | 4 | 4 | 0 |
| 7 | 0 | 3 | 0 | 4 | 3 | 0 |
| 8 | 1 | 3 | 1 | 4 | 4 | 0 |
| 9 | 0 | 4 | 1 | 4 | 4 | 0 |
| 10 | 0 | 3 | 0 | 3 | 3 | 1 |
| Average | 0.3 | 3.2 | 0.3 | 3.8 | 3.7 | 0.2 |
| SD | 0.5 | 0.8 | 0.5 | 0.4 | 0.5 | 0.4 |
| Dunnett's test | vs | *p<0.05 | p>0.05 | *p<0.05 | *p<0.05 | p>0.05 |

| **Damaged cell** | 75 mmHg | 75 mmHg | 75 mmHg | 75 mmHg | 75 mmHg | 75 mmHg |
| --- | --- | --- | --- | --- | --- | --- |
|  |  | 10 μM Vori | 10 μM Vori+ 24SH(1) | 10 μMVori + PTX | 10 μM Vori + APV | 10 μM Vori+ AlloP(1) |
| 1 | 22 | 66 | 0 | 96 | 83 | 1 |
| 2 | 17 | 54 | 2 | 69 | 75 | 2 |
| 3 | 19 | 57 | 1 | 59 | 69 | 0 |
| 4 | 10 | 43 | 0 | 63 | 57 | 3 |
| 5 | 25 | 51 | 4 | 65 | 63 | 2 |
| 6 | 17 | 67 | 3 | 73 | 70 | 2 |
| 7 | 15 | 87 | 5 | 79 | 92 | 5 |
| 8 | 18 | 51 | 1 | 88 | 66 | 0 |
| 9 | 15 | 43 | 1 | 58 | 78 | 1 |
| 10 | 11 | 56 | 3 | 65 | 65 | 3 |
| Average | 16.9 | 57.5 | 2.0 | 71.5 | 71.8 | 1.9 |
| SD | 4.6 | 13.1 | 1.7 | 12.6 | 10.4 | 1.5 |
| Dunnett's test | vs | *p<0.05 | *p<0.05 | *p<0.05 | *p<0.05 | *p<0.05 |

**Source data of Supplementary Table 5.**

| NFLT | 75 mmHg | 75 mmHg+ 1 μM AlloP | 75 mmHg+ 0.2 μM AlloP | 75 mmHg+ 0.1 μM AlloP | 75 mmHg+ 1 μM 24SC | 75 mmHg+ 0.2 μM 24SC | 75 mmHg+ 0.1 μM 24SC | 75 mmHg+ 0.1 μM AlloP+ 0.1 μM 24SC | 75 mmHg+ 0.05 μM AlloP+ 0.05 μM 24SC |
| --- | --- | --- | --- | --- | --- | --- | --- | --- | --- |
| 1 | 11.8 | 2.1 | 2.0 | 6.6 | 2.0 | 1.8 | 7.9 | 2.3 | 13.0 |
| 2 | 11.2 | 1.7 | 1.1 | 3.7 | 1.8 | 1.5 | 8.0 | 2.5 | 12.1 |
| 3 | 12.0 | 1.9 | 2.0 | 4.5 | 1.8 | 1.3 | 10.5 | 2.1 | 11.2 |
| 4 | 12.1 | 2.2 | 1.0 | 4.5 | 2.0 | 2.1 | 7.3 | 2.5 | 10.2 |
| 5 | 11.9 | 1.0 | 1.2 | 5.1 | 1.3 | 1.9 | 3.0 | 2.0 | 10.0 |
| 6 | 11.2 | 0.9 | 1.3 | 2.3 | 2.3 | 1.5 | 1.5 | 1.8 | 11.3 |
| 7 | 9.9 | 2.0 | 1.8 | 4.5 | 1.6 | 1.4 | 4.1 | 1.9 | 10.0 |
| 8 | 12.5 | 1.7 | 1.5 | 3.4 | 1.7 | 2.3 | 3.8 | 2.2 | 13.0 |
| 9 | 10.7 | 1.8 | 1.3 | 3.8 | 2.4 | 2.0 | 5.9 | 1.3 | 11.0 |
| Average | 11.5 | 1.7 | 1.5 | 4.3 | 1.9 | 1.8 | 5.8 | 2.1 | 11.3 |
| SD | 0.8 | 0.5 | 0.4 | 1.2 | 0.3 | 0.3 | 2.9 | 0.4 | 1.2 |
| Dunnett's test | vs | *p<0.05 | *p<0.05 | *p<0.05 | *p<0.05 | *p<0.05 | *p<0.05 | *p<0.05 | p>0.05 |

|  |  |  |  |  |  |  |  |  |  |
| --- | --- | --- | --- | --- | --- | --- | --- | --- | --- |
| NDS | 75 mmHg | 75 mmHg+ 1 μM AlloP | 75 mmHg+ 0.2 μM AlloP | 75 mmHg+ 0.1 μM AlloP | 75 mmHg+ 1 μM 24SC | 75 mmHg+ 0.2 μM 24SC | 75 mmHg+ 0.1 μM 24SC | 75 mmHg+ 0.1 μM AlloP+ 0.1 μM 24SC | 75 mmHg+ 0.05 μM AlloP+ 0.05 μM 24SC |
| 1 | 1 | 0 | 0 | 1 | 0 | 0 | 1 | 0 | 0 |
| 2 | 1 | 0 | 1 | 0 | 0 | 1 | 1 | 0 | 1 |
| 3 | 1 | 0 | 0 | 0 | 0 | 0 | 1 | 0 | 1 |
| 4 | 1 | 1 | 0 | 1 | 1 | 0 | 0 | 0 | 1 |
| 5 | 1 | 0 | 0 | 0 | 1 | 0 | 1 | 0 | 0 |
| 6 | 0 | 0 | 0 | 0 | 0 | 0 | 1 | 0 | 1 |
| 7 | 1 | 0 | 0 | 0 | 0 | 1 | 0 | 1 | 1 |
| 8 | 1 | 0 | 0 | 0 | 0 | 0 | 1 | 0 | 1 |
| 9 | 0 | 0 | 0 | 1 | 0 | 0 | 1 | 0 | 1 |
| Average | 0.8 | 0.1 | 0.1 | 0.3 | 0.2 | 0.2 | 0.8 | 0.1 | 0.8 |
| SD | 0.4 | 0.3 | 0.3 | 0.5 | 0.4 | 0.4 | 0.4 | 0.3 | 0.4 |
| Dunnett's test | vs | *p<0.05 | *p<0.05 | p>0.05 | *p<0.05 | *p<0.05 | p>0.05 | *p<0.05 | p>0.05 |

| Damaged cell | 75 mmHg | 75 mmHg+ 1 μM AlloP | 75 mmHg+ 0.2 μM AlloP | 75 mmHg+ 100 nM AlloP | 75 mmHg+ 1 μM 24SC | 75 mmHg+ 0.2 μM 24SC | 75 mmHg+ 0.1 μM 24SC | 75 mmHg+ 0.1 μM AlloP+ 0.1 μM 24SC | 75 mmHg+ 0.05 μM AlloP+ 0.05 μM 24SC |
| --- | --- | --- | --- | --- | --- | --- | --- | --- | --- |
| 1 | 15 | 1 | 4 | 5 | 2 | 1 | 10 | 2 | 16 |
| 2 | 19 | 2 | 0 | 2 | 2 | 0 | 9 | 1 | 17 |
| 3 | 17 | 4 | 2 | 5 | 1 | 3 | 13 | 2 | 17 |
| 4 | 26 | 1 | 1 | 4 | 0 | 2 | 7 | 0 | 17 |
| 5 | 13 | 2 | 3 | 5 | 4 | 1 | 7 | 1 | 21 |
| 6 | 17 | 0 | 2 | 3 | 3 | 4 | 5 | 2 | 19 |
| 7 | 22 | 1 | 2 | 5 | 2 | 1 | 9 | 1 | 22 |
| 8 | 15 | 0 | 1 | 7 | 1 | 1 | 12 | 2 | 14 |
| 9 | 14 | 3 | 0 | 4 | 0 | 2 | 11 | 3 | 15 |
| Average | 17.6 | 1.6 | 1.7 | 4.4 | 1.7 | 1.7 | 9.2 | 1.6 | 17.6 |
| SD | 4.2 | 1.3 | 1.3 | 1.4 | 1.3 | 1.2 | 2.6 | 0.9 | 2.7 |
| Dunnett's test | vs | *p<0.05 | *p<0.05 | *p<0.05 | *p<0.05 | *p<0.05 | *p<0.05 | *p<0.05 | p>0.05 |

**Source data of Supplementary Table 6-1.**

| **NDS** | 10 mmHg | 10 mmHg | 10 mmhg | 10 mmhg |
| --- | --- | --- | --- | --- |
|  | aCSF | 1 μM Duta | 10 μM Vor | 10 μM Vor + APV + CNQX |
| 1 | 0 | 0 | 4 | 1 |
| 2 | 0 | 0 | 4 | 0 |
| 3 | 1 | 1 | 4 | 1 |
| Average | 0.3 | 0.3 | 4.0 | 0.7 |
| SD | 0.0 | 0.0 | 0.0 | 0.7 |
| Dunnett's test | vs | p>0.05 | *p<0.05 | p>0.05 |
|  |  |  |  |  |
| **NDS** | 10 mmHg | 10 mmhg | 10 mmhg |  |
|  | 10 μM Vor+ 24SH(30) | 10 μM Vor + 24SH(30)+PTX | 10 μM Vor + +AlloP (10) |  |
| 1 | 1 | 4 | 1 |  |
| 2 | 0 | 4 | 0 |  |
| 3 | 1 | 4 | 0 |  |
| Average | 0.7 | 4.0 | 0.3 |  |
| SD | 0.6 | 0.0 | 0.6 |  |
| Dunnett's test | vs | *p<0.05 | p>0.05 |  |

**Source data of Supplementary Table 6-2.**

| **NDS** | 75 mmHg | 75 mmHg | 75 mmHg |
| --- | --- | --- | --- |
|  | 1 μM Duta | 1 μM Duta +APV | 1 μM Duta + AlloP(1) |
| 1 | 4 | 1 | 1 |
| 2 | 4 | 2 | 1 |
| 3 | 4 | 1 | 0 |
| Average | 4.0 | 1.3 | 0.7 |
| SD | 0.0 | 0.6 | 0.6 |
| Dunnett's test | vs | *p<0.05 | *p<0.05 |
|  |  |  |  |
|  |  |  |  |
| **NDS** | 75 mmHg | 75 mmHg | 75 mmHg |
|  | 10 μM Vor | 10 μM Vori+ 24SH(1) | 10 μM Vori+ AlloP (1) |
| 1 | 4 | 0 | 0 |
| 2 | 4 | 1 | 1 |
| 3 | 4 | 2 | 0 |
| Average | 4.0 | 1.0 | 0.3 |
| SD | 0.0 | 1.0 | 0.6 |
| Dunnett's test | vs | *p<0.05 | *p<0.05 |

| **NDS** | 75 mmHg | 75 mmhg |
| --- | --- | --- |
|  | 0.1 μM AlloP | 24SH(0.1)+AlloP (0.1) |
| 1 | 1 | 0 |
| 2 | 1 | 0 |
| 3 | 2 | 0 |
| Average | 1.3 | 0.0 |
| SD | 0.6 | 0.0 |
| Wilcoxon-Mann-Whitney's test | vs | *p<0.05 |
